# Recurrent pulmonary infection, a vicious cycle of multifactorial interactions, and prevention/treatment strategies

**DOI:** 10.3389/fimmu.2026.1945273

**Published:** 2026-10-29

**Authors:** Chaojie Shen, Yuhuan Li, Zongjun Xiang, Min Zhao, Jingsong Wang, Xutao Wang

**Affiliations:** 1 Department of Pharmacy, Jiaxing Hospital of Traditional Chinese Medicine, Jiaxing, Zhejiang, China; 2 Department of Pharmacy, Jiaxing Hospital of Traditional Chinese Medicine Affiliated to Jiaxing University, Jiaxing, Zhejiang, China; 3 Department of Pharmacy, Guangyuan Central Hospital, Guangyuan, Sichuan, China; 4 Department of Obstetrics and Gynecology, The Second People's Hospital of Lizhou District, Guangyuan, Sichuan, China

**Keywords:** host−pathogen interaction, immune evasion, immunomodulation, recurrent pulmonary infection, risk factors, vaccination

## Abstract

Recurrent pulmonary infection (RPI) refers to the frequent and repeated episodes of lower respiratory tract infections. At its core, it represents a vicious cycle arising from the disruption of the homeostatic balance among pathogens, host immunity, and pulmonary architecture. RPI accounts for a considerable proportion of pediatric pneumonia cases, and the cumulative incidence among elderly patients hospitalized for pneumonia is also markedly elevated, imposing a substantial disease burden. The etiologies are notably diverse, beyond common pathogens such as bacteria and viruses, the ubiquity of asymptomatic colonization, underlying comorbidities, immune deficiencies, medication-related factors, and aspiration all constitute critical predisposing factors. This review summarizes the pathogenic characteristics of RPI, host-related factors, and the mechanistic roles of structural lung damage in infection recurrence. It further focuses on the current advances and limitations of precision diagnostic approaches (metagenomic sequencing), immunomodulatory and microbiome-targeted interventions, and vaccination strategies. Existing evidence suggests that the clinical benefits of these interventions in RPI remain to be validated by more high-quality studies. The aim of this article is to provide a reference for the clinical management of RPI and to inform future research directions.

## 1 Introduction

Recurrent pulmonary infection (RPI) refers to the frequent and repeated episodes of lower respiratory tract infections. In clinical research, RPI encompasses two main scenarios. One is recurrent pneumonia, defined as at least two episodes of pneumonia within one year, or at least three episodes of pneumonia ever, with intercritical radiographic clearing of densities (1). The other is recurrent lower respiratory tract infection (rLRTI), which refers to repeated symptoms of lower respiratory tract infection but does not necessarily have radiographic evidence of pneumonia (2). It is particularly important to note that recurrent clinical events may be caused by two different mechanisms, namely relapse of the same pathogen or reinfection by a new pathogen (3, 4). The former usually reflects incomplete treatment or endogenous reactivation, whereas the latter suggests persistently impaired host defense or re-exposure to the pathogen environment. In addition, simple asymptomatic colonization and chronic persistent infection do not fall within the core scope of RPI, but both may be discussed as important risk factors or consequences in the pathogenesis of RPI (5).

Pneumonia, as the underlying disease of RPI, is an acute pulmonary infection caused by microorganisms such as bacteria, viruses, fungi, or parasites. The pathogens invade the pulmonary parenchyma through respiratory inhalation, hematogenous dissemination, or spread from adjacent tissues. Each year, millions of people worldwide contract pneumonia, imposing a heavy burden on public health systems. In regions with limited medical resources, pneumonia remains one of the leading causes of death (6–8). The human body possesses multiple defense mechanisms against respiratory infections, including physical barriers such as nasal hair filtration, mucociliary clearance, and epithelial integrity, along with humoral immunity mediated by the complement system and cellular immunity involving phagocytes (9, 10). Nevertheless, these defenses can be breached in certain susceptible populations, leading to pulmonary infection that typically manifests as acute lower respiratory tract symptoms (11). The persistent presence of pulmonary pathogens, host immune deficiency, and structural damage to lung tissue interact to form a vicious cycle of infection, lung injury, and reinfection, which manifests as repeated episodes of lower respiratory tract infection (12–14). RPI is not a single disease entity but rather a common clinical endpoint arising from multiple etiologies. Its risk factors are distinctive and include extreme age—namely children under 5 years and adults over 65 years—the presence of structural lung disease or immunodeficiency, and exposure to healthcare settings that predispose to drug-resistant organisms (15, 16). Of note, impaired lung development following childhood pneumonia can lead to a lifelong state of susceptibility, often manifesting as restrictive lung disease, bronchiectasis, and other sequelae (17, 18). In elderly patients, repeated infections due to comorbidities markedly increase the risk of fatal complications such as sepsis and acute respiratory distress syndrome (ARDS) (19, 20).

Epidemiological data indicate that the prevalence of RPI varies significantly across different populations, and the etiological composition is distinctive in each group. Among children, RPI accounts for 7.7% to 9.0% of pediatric pneumonia cases, and this proportion rises to 14.4% in children with recurrent lower respiratory tract infections (21). The causes are predominantly recurrent aspiration, congenital airway structural abnormalities, impaired airway clearance, and immunodeficiency (22, 23). In adults, the recurrence rate of RPI among patients discharged after community-acquired pneumonia (CAP) ranges from 9% to 12% within 3 to 5 years, with a median time to recurrence of 123 to 317 days and an associated mortality of 4% to 10% (24). The etiologies more often involve acquired immunosuppression, bronchiectasis, and chronic obstructive pulmonary disease (25). In hospitalized elderly pneumonia patients aged 75 years or older, the 3-year cumulative incidence of RPI reaches as high as 35.2% (26), and immunosenescence and age-related organ functional decline are risk factors for recurrence that are independent of underlying diseases (27, 28). In patients with cystic fibrosis, altered mucus physicochemical properties and *Pseudomonas aeruginosa* biofilm formation caused by *CFTR* gene mutations constitute the core drivers of chronic infection and recurrent acute exacerbations (29, 30).

The clinical management of RPI faces multiple challenges in diagnosis, treatment, and prevention. In terms of diagnosis, pathogen identification is often complicated by prolonged antibiotic exposure and the confounding presence of colonizing organisms versus true pathogens in the respiratory tract (31). Regarding treatment, empirical anti-infective strategies are hampered by the difficulty of pinpointing the causative agent, and antimicrobial resistance is becoming increasingly problematic (32). As for prevention, there is currently no unified standard for long-term management strategies in high-risk populations (24). Therefore, beyond acute-phase therapy, there is an urgent need to establish long-term surveillance systems for susceptible individuals, with a particular focus on pulmonary function rehabilitation and immunomodulation, so as to interrupt the vicious cycle of recurrent infection. This review analyzes RPI caused by multiple factors, explores its mechanisms, risk factors, and prevention and treatment strategies, and aims to provide a reference for clinical management and future research.

## 2 Causes of recurrent pulmonary infection

RPI is not uncommon among patients who have apparently recovered from pneumonia. Its pathogenesis involves multiple interrelated levels. The destruction of pulmonary tissue architecture caused by acute infection directly impairs local defense mechanisms (33, 34). Meanwhile, the selective pressure imposed by prolonged antibiotic use promotes the emergence of drug-resistant organisms, thereby substantially increasing the risk of reinfection (10). Concurrent underlying conditions such as chronic obstructive pulmonary disease and heart failure, together with immunosuppressive states including drug-induced immune suppression, further compromise host resistance in a sustained manner (35). The interplay between recurrence and tissue damage resulting from these factors may eventually give rise to irreversible lesions, including bronchiectasis and chronic suppurative lung disease (36). From a demographic perspective, RPI predominantly affects infants and young children, the elderly, patients with impaired pulmonary function, and those receiving corticosteroids or antimicrobial therapy (14, 37). Overall, although recurrent infections are directly provoked by microorganisms, underlying diseases, structural lung damage, and drug-related factors are equally critical permissive conditions that perpetuate these repeated episodes.

### 2.1 Role of microbial factors in recurrent pulmonary infection

The lung is particularly susceptible to infection owing to its continuous exposure to environmental microorganisms and its unique anatomical structure (38). The development of RPI is closely linked to the ability of specific pathogens to breach host defenses and establish persistent colonization or repeated invasion.

From an epidemiological standpoint, the microbiological profile of RPI varies considerably across different populations. An observational cohort study that enrolled 1556 hospitalized CAP patients identified 146 cases of recurrent CAP, accounting for 9.4% of the entire cohort. Compared with patients who experienced a single pneumonia episode, those with recurrent pneumonia showed significantly higher detection rates of *Haemophilus* influenzae and other Gram-negative bacilli (32). In certain high-risk groups, the predominance of particular pathogens becomes even more prominent. Among patients with cystic fibrosis (CF), chronic infection with *Pseudomonas aeruginosa* consistently serves as a core driver of declining lung function and acute exacerbations, with infection rates exceeding 50% in adult CF patients (39, 40). In pediatric RPI, the contribution of viruses is by no means negligible. A 13-year retrospective study demonstrated that viral pathogens were detectable in 64.3% of children with recurrent pneumonia, human rhinovirus was the most common, accounting for 30.5%, followed by adenovirus at 17.2% and respiratory syncytial virus at 13.9% (41). In the general population, *Streptococcus pneumoniae* remains the most important bacterial pathogen in CAP (42). Furthermore, tuberculosis recurrence rates exhibit marked regional variations, and the relative contributions of reinfection versus reactivation differ according to local epidemiological backgrounds (43, 44). Collectively, these observations indicate that the microbiological composition of RPI is highly specific, with the predominance of particular pathogens being determined by the host’s underlying diseases and immune status. Meanwhile, pathogens can promote recurrent episodes through a variety of mechanisms, including immune evasion (45), antigenic variation (46), biofilm formation (47), and the induction of immunopathological injury (48).

#### 2.1.1 Bacterial pathogens and recurrent pulmonary infection

A variety of bacteria can drive recurrent pulmonary infections through mechanisms such as immune evasion, acquisition of antimicrobial resistance, and persistent colonization within the host.

The capsular polysaccharide of *Streptococcus pneumoniae* constitutes its core virulence determinant. Based on structural differences in the capsular polysaccharide, this organism can be divided into more than 100 serotypes (49). Notably, asymptomatic nasopharyngeal colonization by *Streptococcus pneumoniae* is extremely common in healthy individuals, particularly in children. A meta-analysis focusing on healthy children in Southeast Asia reported a pooled carriage rate of 36.0% (95% CI 34.2%–37.8%) among those under 5 years of age, with marked intercountry variation ranging from 68.0% (95% CI 61.9%–74.0%) in Cambodia to 7.6% (95% CI 5.7%–9.6%) in Malaysia (50). In sub-Saharan Africa, the carriage rate in children under 5 years is even higher, reaching 63.2% (95% CI 55.6–70.8) (51). After birth, nasopharyngeal colonization rates rise rapidly, approaching approximately 77% by 2 months of age and 86% by 5 months (52). In low-income countries, the pooled carriage rate of *Streptococcus pneumoniae* among children under 5 years before the introduction of pneumococcal conjugate vaccine (PCV) was estimated at 64.8% (95% CI 49.8%–76.1%) (53). Anti-capsular antibodies generated after primary infection are serotype-specific and predominantly confer protection against the particular capsular type of the infecting strain, though some degree of cross-immunity does exist between different serotypes. Capsular polysaccharides with similar structures, especially those within the same serogroup, can induce cross-reactive antibodies. However, this cross-protection tends to be limited and incomplete (49). On the other hand, several conserved protein antigens expressed by *Streptococcus pneumoniae*, such as PspA, are highly shared across different serotypes and have been demonstrated to elicit protective responses against heterologous capsular serotypes (54). In addition, CD4-positive T-cell responses, particularly those mediated by Th17 cells, can confer serotype-independent protection (55, 56). Nevertheless, because of incomplete cross-reactivity of capsular polysaccharides and interindividual variation in immune response efficacy, such cross-protection is often insufficient to completely prevent colonization and reinfection by heterotypic *Streptococcus pneumoniae*, and this constitutes an important immunological basis for recurrent pneumonia caused by different serotypes (57).

*Haemophilus* influenzae is a significant pathogen in community-acquired pneumonia and is also a common colonizer of the upper respiratory tract in healthy children. The nasopharyngeal carriage rate of H. influenzae among healthy Chinese children is approximately 21% (58). Another systematic review covering healthy Chinese populations reported a pharyngeal carriage rate of 17% (95% CI 13%–21%) in children (59). In bronchoalveolar lavage fluid from children with recurrent community-acquired pneumonia, non-typeable *Haemophilus influenzae* (NTHi) was identified as one of the predominant pathogens, with a detection rate as high as 51.2%, suggesting that colonization by this organism is closely associated with repeated pneumonia episodes (60). The host immune response to NTHi is frequently inadequate to clear pulmonary bacteria, which leads to recurrent or persistent infection and triggers chronic inflammation and lung tissue damage (61).

*Mycoplasma pneumoniae* is a common causative agent of community-acquired pneumonia, and its asymptomatic carriage in children should not be overlooked. Observational studies have shown that the PCR detection rate of *Mycoplasma pneumoniae* DNA in asymptomatic children can reach 21.2% (95% CI 17.2%–25.2%) (62). Among children with recurrent respiratory tract infections, the carriage rate of *Mycoplasma pneumoniae* is as high as 68%, and carriers exhibit lower microbial diversity and a higher proportion of disease-associated microbial community members than non-carriers (63). Furthermore, up to 56% of asymptomatic children can carry two or more pathogens simultaneously (62). Such a high prevalence of asymptomatic carriage suggests that a single positive PCR result should be interpreted with caution in the etiological diagnosis of recurrent pneumonia.

*Staphylococcus aureus* is a common colonizer of the human upper respiratory tract and skin, with approximately 30% of the population being persistent nasal carriers (64). However, when host defense barriers are compromised or immune function is impaired, this organism can transition from a commensal state to an opportunistic pathogen, causing severe infections including pneumonia (65). In respiratory infections, *Staphylococcus aureus* is an important pathogen in both hospital-acquired and community-acquired pneumonia and is closely associated with recurrent acute pulmonary exacerbations and progressive decline in lung function in patients with cystic fibrosis (65, 66). Colonization is an important prerequisite for recurrent infection. Studies have shown that previous methicillin-resistant *Staphylococcus aureus* (MRSA) infection or colonization is an independent risk factor for MRSA pneumonia (OR 6.21, 95% CI 3.25–11.85) (67). The propensity of *Staphylococcus aureus* to cause recurrent infection is intimately linked to its multiple immune evasion strategies (68). This organism secretes a variety of immune-evasive molecules, including staphylococcal protein A, chemotaxis inhibitory protein, and leukocidins, which interfere with neutrophil chemotaxis, phagocytosis, and killing, thereby impairing the host’s ability to clear the pathogen (69). Moreover, *Staphylococcus aureus* can form small colony variants, a phenotype characterized by reduced metabolic activity and slow growth that allows the bacteria to survive intracellularly for prolonged periods, thus escaping immune surveillance and antibiotic killing, and representing a major cause of chronicity and recurrent episodes (70, 71). Biofilm formation constitutes another important mechanism. By producing an extracellular matrix of polysaccharides and proteins, bacteria establish biofilms that physically shield them from antibiotic penetration and protect them from immune cell attack. Biofilm-associated infections often lead to symptom relapse after antibiotic courses are completed (72). As a resistant subtype of Staphylococcus aureus, MRSA can cause severe pulmonary necrosis and inflammatory injury, and its infections frequently take a fulminant course with high mortality (65, 73, 74). In summary, the persistent presence of *Staphylococcus aureus* in the respiratory tract and its tendency to cause recurrent infection result from the combined action of potent immune evasion, biofilm formation, and the small colony variant phenotype (69, 71).

*Pseudomonas aeruginosa* achieves long-term survival within the host by forming biofilms on mucosal surfaces and embedding into deeper structures, which is the core mechanism underlying its recurrent infection episodes (75). The emergence of multidrug-resistant strains further increases the difficulty of treating RPI in immunocompromised and hospitalized patients (75). In patients with cystic fibrosis in particular, impaired immune function combined with the airway environment jointly facilitates repeated *Pseudomonas aeruginosa* infections, which often progress to refractory chronic pulmonary infection (76, 77).

*Mycobacterium tuberculosis* is one of the most harmful respiratory pathogens, and tuberculosis has an extremely high recurrence rate and is an important cause of RPI. Recurrence can be caused by exogenous reinfection or endogenous reactivation of latent foci, and residual lung tissue damage from the primary infection can persistently increase the risk of recurrence (78). The likelihood of tuberculosis recurrence is closely associated with the completeness of initial treatment, the host immune status, and the risk of re-exposure to the pathogen in the environment (79, 80). In regions with high tuberculosis endemicity, BCG vaccination is an important public health measure for reducing the incidence of tuberculosis and the risk of latent infection, but the widespread occurrence of exogenous reinfection limits its protective effect (81) (82). The recurrence rate in patients with multidrug-resistant tuberculosis is significantly higher than that in patients with drug-susceptible tuberculosis (83). A retrospective cohort study of multidrug-resistant tuberculosis patients reported that 34.3% experienced recurrence (84). Among recurrent tuberculosis cases, approximately 11% acquire new drug resistance during treatment, with fluoroquinolone resistance being the most common (85). Therefore, in high-tuberculosis-prevalence areas, implementing standardized BCG vaccination and strengthening surveillance and management of drug-resistant tuberculosis are of great significance for reducing tuberculosis recurrence rates.

#### 2.1.2 Pathogens and recurrent pulmonary infection

Viruses constitute an important group of pathogens responsible for recurrent pulmonary infections, and their role is particularly prominent in pediatric populations. The mechanisms by which viral infections lead to recurrence operate at multiple levels. Viruses can interfere with the maturation and migration of dendritic cells as well as T-cell activation, thereby compromising the host’s ability to establish protective immunity (86). Defects in virus-specific memory T-cell function and impaired recall responses permit clinical disease to occur even in the presence of pre-existing immunity (87, 88). Moreover, viruses can continuously evade established humoral immune responses through mechanisms such as antigenic drift (89). Infections with respiratory syncytial virus (RSV) and human metapneumovirus (hMPV) can also trigger aberrant T-cell responses that exacerbate pulmonary inflammation and lead to poor establishment of T- and B-cell immune memory (90). Collectively, these mechanisms form the immunological foundation for recurrent viral pulmonary infections.

RSV and rhinovirus (RV) are the most important respiratory viral pathogens in childhood. These two viruses not only constitute major risk factors for wheezing and asthma in infants and young children (91) but are also closely linked to frequent infection recurrence, with RV being the most common trigger of acute asthma exacerbations in children (92). It should be noted that RV can also be detected in asymptomatic infants, and such early infections can alter the respiratory immune environment, which is associated with increased susceptibility to subsequent infections (93). Specifically, early RV infection can modify the host’s immune response pattern to subsequent heterotypic viral infections, inducing an asthma-like phenotype characterized by type 2 inflammation, mucous metaplasia, and eosinophilic infiltration (94). This effect is mediated by type 2 innate lymphoid cells (ILC2), whose development depends on retinoic acid receptor-related orphan receptor α (93). At the cellular level, repeated RV infections can induce aberrantly amplified innate immune responses in airway epithelial cells, manifesting as enhanced expression of inflammatory factors such as RIG-I and IL-8, and after exposure to IL-13, these cells release greater amounts of inflammatory mediators including IL-4 and IL-5 (95, 96). Concurrently, RV infection disrupts the tight junction structures of the airway epithelium, leading to damage to ZO-1-positive tight junctions, reduced transepithelial electrical resistance, and increased barrier permeability (97). This barrier dysfunction creates conditions favorable for penetration by allergens and pathogens, further increasing susceptibility to subsequent infections (95).

Asymptomatic carriage is by no means unique to rhinovirus but is rather a general feature of respiratory viruses. Research has shown that detectable respiratory viruses are present in 28% to 41% of asymptomatic children (98–100). The rates of asymptomatic detection vary among different viruses, and viral carriage without symptoms is more common in younger children. However, a positive virus detection during an asymptomatic period itself is associated with a lower risk of subsequent acute respiratory infection, whereas increased nasopharyngeal density of Streptococcus pneumoniae during that same period is significantly correlated with a higher risk of subsequent acute respiratory infection (p< 0.001) (101). This finding suggests that viruses may indirectly raise the risk of infection recurrence by increasing the colonization density of opportunistic bacterial pathogens. Therefore, when interpreting the pathogenic significance of viruses in recurrent pulmonary infection, one must integrate viral load, host immune status, and clinical context into the overall assessment.

The coronavirus disease 2019 (COVID-19), caused by severe acute respiratory syndrome coronavirus 2 (SARS-CoV-2), has emerged as a global health threat. As new variants with immune-evasive capabilities continue to appear, reinfection with SARS-CoV-2 has become increasingly common (102, 103). The mechanisms underlying reinfection involve the dual challenge that viral variants pose to both innate and adaptive immune responses. Under the selective pressure exerted by the human population, the virus undergoes continuous evolution, coupled with the natural waning of immune levels following individual infection or vaccination, this facilitates the dominance of immune-escape variants and thereby substantially elevates the risk of reinfection (104).

Recurrent epidemics and infections with seasonal influenza viruses represent another important cause of recurrent pulmonary infection. High transmission efficiency is certainly a contributing factor, but the root cause lies in the virus’s ability to achieve immune evasion through continuous antigenic drift and antigenic shift (105). Emerging viral strains can effectively evade the immunity established by previous seasonal strains through either infection or vaccination, leaving hosts vulnerable to repeat infections with the same viral subtype across different epidemic seasons (106).

#### 2.1.3 Fungal pathogens and recurrent pulmonary infection

Recurrence of fungal pulmonary infections is closely linked to the latent colonization capacity of the pathogens and the host’s immune status. The core mechanisms involve iatrogenic factors that induce immunosuppression and dysbiosis, as well as the reactivation of pathogens that have established latent infection within the host.

*Candida albicans* is an important opportunistic fungal pathogen that can proliferate excessively in the setting of dysbiosis caused by broad-spectrum antibiotic use, which is an important condition for its role in recurrent pulmonary infection (107). The administration of immunosuppressive agents such as glucocorticoids further suppresses host immune function and significantly increases the risk of invasive infection and recurrence (108).

Infection with *Cryptococcus neoformans* begins with inhalation of environmental spores or yeast cells. Once the pathogen reaches the alveoli, it can be phagocytosed by macrophages but is not killed. Instead, it establishes a latent infection. Once host immunity declines, the latent pathogen can reactivate and replicate and disseminate intracellularly, which is the core mechanism leading to recurrent infection (109, 110).

The clinical manifestations of *Aspergillus* infection are often atypical, and patients may present without fever or obvious respiratory symptoms. This can easily lead to missed or delayed diagnosis, thereby creating conditions for persistent infection and subsequent recurrence (111, 112).

#### 2.1.4 Mixed pathogen infections and recurrent pulmonary infection

RPI often results from the synergistic or sequential actions of multiple pathogens rather than from a single microorganism alone. Interactions among different pathogens can disrupt respiratory immune homeostasis and exacerbate tissue damage, thereby substantially increasing the risk of infection recurrence and complicating clinical management.

Respiratory viral infections serve as a major trigger for secondary bacterial pneumonia, and the underlying mechanisms operate at several levels. Viral infection can damage the airway epithelial barrier, inhibit mucociliary clearance, and downregulate the production of type I interferons as well as the expression of interferon-stimulated genes, thus markedly enhancing host susceptibility to bacterial pathogens (113, 114). During influenza epidemics, co-infections with viruses and bacteria are particularly common, with *Streptococcus pneumoniae* and *Staphylococcus aureus* being the most frequently identified co-pathogens (115). This mechanistic link also partly explains why empirical antibiotics are often administered in viral recurrent infections in clinical practice to prevent severe bacterial complications (116).

COVID-19 is also frequently complicated by mixed infections. Evidence has confirmed that co-infection with SARS-CoV-2 and other pathogens such as influenza A virus can exert synergistic pathogenic effects and aggravate disease severity. Such mixed infections not only increase the difficulty of differential diagnosis but are also significantly associated with prolonged hospital stays and elevated mortality risk (117–119).

Tuberculosis, as a chronic granulomatous infection, causes structural destruction of lung tissue and induces local immune defects, thereby creating favorable conditions for colonization and secondary infection by bacteria such as *Streptococcus pneumoniae* and *Haemophilus* influenzae, as well as by fungi like *Aspergillus*. A history of tuberculosis not only carries its own risk of recurrence but also significantly increases the probability of subsequent bacterial and fungal pulmonary infections, giving rise to a complex and intertwined infectious picture (120).

### 2.2 Lung tissue injury and recurrent infection

#### 2.2.1 Acute infection and immunopathological injury

The immune defense of the lung constitutes a complex and finely tuned system. When pathogens invade, an uncontrolled immune response can trigger excessive inflammation, which in turn directly damages lung tissue (121). This pathological process operates primarily through neutrophil- and platelet-dependent pathways that disrupt the pulmonary endothelial and epithelial barriers (122–124). In the setting of severe infection, pathogens themselves can directly destroy the pulmonary parenchyma and impair alveolar gas exchange, leading to serious sequelae such as respiratory failure while simultaneously establishing the pathological groundwork for recurrence (125, 126). In pediatric populations, acute infection may also interfere with normal lung development, thereby creating a long-term state of susceptibility to pneumonia (18).

If acute lung injury persists, it can give rise to a variety of irreversible structural remodeling processes within the lung. Beyond bronchiectasis, these include bronchiolitis obliterans, characterized by inflammatory narrowing and occlusion of terminal bronchioles, and pulmonary fibrosis, marked by thickening of the alveolar septa and reduced lung compliance (126–128). Taking bronchiectasis as an example, its pathogenesis involves the combined effects of multiple factors, including microbial dysbiosis, aberrant host immune responses, impaired mucociliary clearance, and structural disruption of the airway wall (12). In this process, neutrophil-predominant hyperinflammation stands out as a central component. It not only causes tissue destruction but also impairs mucociliary function, thereby perpetuating a continuous cycle of infection, inflammation, and injury that substantially elevates the risk of acute exacerbations and infection recurrence (12, 129).

#### 2.2.2 Systemic effects and susceptibility to infection

Severe pulmonary infection can progress to sepsis, leading to lung dysfunction and multiorgan failure (126). Whether caused by bacteria or viruses such as influenza A virus, sepsis inflicts substantial damage on pulmonary tissue (130, 131). Viral sepsis is characterized by a dysregulated host response to viral infection, manifesting as early hyperinflammation and subsequent immunosuppression that may occur sequentially or coexist (132). During the immunosuppressive phase, T-cell dysfunction is particularly prominent. Recurrent sepsis can aggravate CD4-positive T-cell exhaustion and concurrently impair antiviral immune responses (133). Survivors of sepsis are left with suppressed immune function, rendering them more susceptible to secondary infections, and their CD8-positive T-cells are more prone to exhaustion (134). In severe sepsis patients who develop cytomegalovirus (CMV) reactivation, the multifunctionality of CD8-positive T-cells is compromised, and this impairment correlates with programmed death-1 (PD-1) expression (135). Clinically, reactivation of latent viruses such as CMV and Epstein-Barr virus (EBV) occurs frequently in sepsis patients, with a frequency comparable to that seen in transplant recipients receiving immunosuppressive therapy, which directly reflects the state of immune suppression induced by sepsis (136). The reactivated viruses can further disseminate to multiple organs throughout the body, exacerbating tissue injury and establishing a vicious cycle between immunosuppression and viral replication (137). Consequently, viral sepsis not only facilitates pathogen dissemination but also markedly increases the risk of infection recurrence. Furthermore, underlying chronic bacterial pulmonary infection can exacerbate pathological inflammation, accelerate the decline of lung function, and undermine host immunity against bacteria, thereby rendering patients more vulnerable to subsequent viral infections and giving rise to new peaks of infectious episodes (138). However, the mechanisms of sepsis and immune exhaustion are largely based on adult critically ill patients and animal models, and their applicability in pediatric and elderly RPI patients requires careful evaluation.

### 2.3 Drug-related factors and recurrent infection

Immunosuppressive agents, including glucocorticoids, calcineurin inhibitors, antimetabolites, tumor necrosis factor (TNF)-α inhibitors, and anti-CD20 monoclonal antibodies, exert their therapeutic effects while broadly attenuating both innate and adaptive host immune responses. Drug-induced immunosuppression can increase the risks of latent infection reactivation and *de novo* opportunistic infections (139, 140). In patients with cryptococcal meningitis, prior glucocorticoid exposure has been associated with an increased 1-year mortality risk (OR 1.3, 95% CI 1.2-2.0) (141). Among children with systemic lupus erythematosus receiving long-term high-dose glucocorticoid therapy, elevated creatinine levels and reduced lymphocyte counts have been identified as independent risk factors for Pneumocystis jirovecii pneumonia (PJP) (142). TNF inhibitors are also linked to an elevated risk of infectious pneumonia; real-world data analyses have revealed positive safety signals for infliximab and golimumab with respect to infectious pneumonia, with tuberculosis-related diseases being the most frequently reported (143). Under these immunosuppressive conditions, patients not only exhibit heightened susceptibility to common pathogens but also face a substantially increased risk of opportunistic infections and recurrent lower respiratory tract infections.

Antibiotic therapy, while eradicating target pathogens, may simultaneously influence subsequent infection recurrence. Broad-spectrum antibiotic use can disrupt the normal microbiota of the respiratory tract and gut, leading to ecological imbalance and creating conditions favorable for colonization and overgrowth by drug-resistant or opportunistic organisms (144). However, the true attributable role of resistant strains in recurrent pulmonary infections remains incompletely understood. Available evidence indicates that pathogens isolated from patients with recurrent respiratory infections exhibit considerable levels of antimicrobial resistance. For instance, *Pseudomonas aeruginosa* shows resistance rates exceeding 30% against 10 antibiotics, and *Klebsiella pneumoniae* displays resistance rates above 35% against 16 antibiotics (144). In patients with severe pneumonia, 71% of uneradicated strains already possessed or acquired resistance to the antibiotics used during treatment, and among 10 patients with recurrence, 7 experienced recurrence linked to uneradicated pathogens (145). The infection recurrence rate in patients with carbapenem-resistant Enterobacteriaceae infections has been reported as 16.8% (146). In chronic pulmonary infections caused by *Pseudomonas aeruginosa*, 83.3% of recurrences were attributed to the same strain (39). Patients with recurrent invasive pneumococcal disease were more likely to have harbored resistance markers at the time of their initial episode (147). Collectively, these findings suggest an association between drug-resistant strains and pulmonary infection recurrence, although the precise attributable proportion and the independent role of resistance in RPI require further investigation.

Irrational or inappropriate antibiotic use imposes strong selective pressure that favors the emergence of resistant strains. Some resistant bacteria can evade antibiotic killing by entering a persister state or forming biofilms, thereby perpetuating the infectious process (148–150). Nevertheless, the extent to which drug-resistant organisms constitute an independent etiology of RPI is currently lacking systematic attribution data.

Certain medications can impair swallowing function or reduce the level of consciousness, and the covert or overt aspiration resulting from these effects represents an important trigger for pulmonary infection. Drugs implicated in this mechanism include sedatives, antipsychotics, and anticholinergic agents. Aspiration syndrome accounts for a considerable proportion of recurrent pneumonia etiologies. A study of 238 hospitalized children with recurrent pneumonia found that 48% had swallowing dysfunction (151). Another investigation encompassing 551 children with recurrent pneumonia identified recurrent aspiration as the most common cause, accounting for 23.0% of cases (152). In adult populations, a Japanese multicenter prospective study reported that aspiration pneumonia occurred in 60.1% of community-acquired pneumonia cases and 86.7% of hospital-acquired pneumonia cases (153). Aspiration pneumonia carries a high recurrence risk. A prospective cohort study of elderly patients hospitalized with pneumonia demonstrated that those receiving professional oral care had a recurrence rate of 27.5%, whereas those without such care experienced a rate of 44.7% (154). Among pneumonia patients with four or more aspiration risk factors—including impaired consciousness, bedridden status, cerebrovascular disease, dementia, sedative use, and gastroesophageal disorders—the recurrence rate reached as high as 54.2% (155). Repeated aspiration events can introduce oropharyngeal pathogens into the lower respiratory tract, serving as a persistent source of recurrent pulmonary infection, particularly with anaerobic and Gram-negative organisms. Nevertheless, evidence related to medications and aspiration is mainly derived from adult and elderly patients, and the risk profile in pediatric RPI may differ.

### 2.4 Disease-related factors and recurrent infection

#### 2.4.1 Primary immunodeficiency diseases

Primary immunodeficiency diseases (PIDs) constitute a group of disorders caused by genetic defects in the development or function of the innate immune system, and they represent an important intrinsic etiology of recurrent pulmonary infections. These diseases are highly heterogeneous in their manifestations, yet they share a common clinical feature of extreme susceptibility to pathogens, particularly to specific categories of microorganisms (156). Severe combined immunodeficiency (SCID) is characterized by profound deficiency or dysfunction of T cells and B cells, and affected infants typically present with recurrent and severe systemic infections early in life (157). Among PIDs, those primarily manifested as antibody production defects are the most common, with common variable immunodeficiency (CVID) being a representative example. Patients with CVID predominantly experience recurrent bacterial infections of the upper and lower respiratory tracts, with encapsulated bacteria such as *Streptococcus pneumoniae* and *Haemophilus* influenzae being the frequently implicated pathogens. These infections tend to become chronic and may lead to bronchiectasis, as well as serious complications including pleuritis and empyema (158, 159). Phagocyte functional defects, such as chronic granulomatous disease (CGD), arise from the inability of phagocytes to effectively generate reactive oxygen species, rendering patients susceptible to frequent bacterial and invasive fungal pulmonary infections (112).

#### 2.4.2 Acquired immunodeficiency states

In contrast to congenital defects, acquired immunodeficiency represents a more prevalent clinical factor contributing to recurrent pulmonary infections. Human immunodeficiency virus (HIV) infection serves as a paradigmatic example; the virus specifically destroys CD4-positive T lymphocytes, thereby inducing acquired cellular immune deficiency and rendering affected individuals susceptible hosts to a wide array of opportunistic pathogens. Bacterial pneumonia remains one of the most common causes of hospitalization in this population (160). Iatrogenic immunosuppression constitutes another critical determinant. Patients receiving chemotherapy for malignancies or undergoing long-term immunosuppressive therapy to maintain organ allografts experience pharmacologically attenuated immune function, which substantially compromises their ability to combat pathogens and consequently leads to a markedly increased risk of recurrent pulmonary infections (161).

#### 2.4.3 Autoimmune diseases

Autoimmune conditions such as primary Sjögren’s syndrome, systemic lupus erythematosus, and rheumatoid arthritis can affect the lung through complex mechanisms and thereby increase the risk of infection recurrence (162). Taking primary Sjögren’s syndrome (pSS) as an example, its pathological manifestations extend far beyond exocrine gland hypofunction. Within the lung, this disease can cause lymphocytic infiltration, viscous airway secretions, impaired mucociliary clearance, and may secondarily give rise to interstitial lung disease (ILD) and structural airway abnormalities including bronchiectasis (163). These pathological alterations collectively disrupt the local immune defense microenvironment of the lung. Consequently, even in the absence of immunosuppressive therapy, patients with pSS frequently experience recurrent pulmonary infections (164).

#### 2.4.4 Other disease-related factors

Structural or functional abnormalities of the lung and other related systems constitute another major direct cause of disrupted host defense barriers and subsequent infection recurrence. Swallowing dysfunction, whether overt or silent aspiration, permits bacteria from oropharyngeal secretions to enter the lower respiratory tract repeatedly, representing an underappreciated but important trigger for recurrent pneumonia across all age groups (165, 166). The perioperative period, particularly following thoracic or abdominal surgery, is associated with pain that inhibits deep breathing and effective coughing, leading to retention of respiratory secretions and creating favorable conditions for pathogen proliferation (167–169). In pediatric populations, the etiological spectrum of recurrent pneumonia exhibits certain distinctive features. Beyond immunodeficiency, recurrent aspiration, congenital heart disease, primary ciliary dyskinesia (PCD), and bronchiolitis obliterans (BO) are among the high-risk conditions that warrant particular attention in differential diagnosis (152). It should be noted that evidence related to immunodeficiency is mainly derived from populations with primary immunodeficiency and HIV infection. Their recurrence mechanisms involve specific immune defects, which are fundamentally different from RPI caused by aging or iatrogenic immunosuppression, so cross-population extrapolation should be cautious.

## 3 Treatment and prevention of recurrent infection

### 3.1 Anti-infective therapeutic strategies

The judicious use of anti-infective agents constitutes the core component in controlling acute infection and interrupting the cycle of recurrence. Clinical initial therapy requires a careful balance between empirical broad-spectrum coverage and subsequent pathogen-directed targeted therapy based on microbiological evidence. The key lies in selecting sensitive antibiotics strictly according to antimicrobial susceptibility testing results, so as to maximize therapeutic efficacy while avoiding the selection of resistant strains (170). Meanwhile, mucoactive agents such as ambroxol may increase the concentrations of various antibiotics in lung tissue and airway secretions, including beta-lactams and macrolides, suggesting their potential as adjunctive antibacterial agents (171). Azithromycin possesses important immunomodulatory properties beyond its antimicrobial effects. In patients with cystic fibrosis, azithromycin has been found to substantially enhance macrophage uptake and killing of *Pseudomonas aeruginosa* and to promote polarization toward the anti-inflammatory M2 macrophage phenotype (172). Some studies suggest that early-life azithromycin administration in children may reduce the risk of severe lower respiratory tract disease (173), but this conclusion still requires validation by more high-quality studies. Mechanistically, azithromycin can inhibit the STAT1 and NF-κB signaling pathways, thereby driving macrophage polarization toward the M2 anti-inflammatory phenotype and reducing the release of pro-inflammatory cytokines, which in turn attenuates the excessive inflammatory response and tissue injury triggered by infection (174). This mechanism may partly explain the potential clinical benefits of azithromycin in reducing acute exacerbations and severe lower respiratory tract infections in patients with chronic respiratory diseases, but it remains a mechanistic inference. However, the risks of long-term macrolide use cannot be ignored. Prolonged use can induce resistant strains, and resistance rates of Streptococcus pneumoniae and *Staphylococcus aureus* to macrolides are already relatively high in some regions, which may weaken their antibacterial efficacy and increase the risk of transmission of resistant bacteria (175). In addition, azithromycin can cause QT interval prolongation and increase the risk of torsades de pointes, particularly in patients with hypokalemia, hypomagnesemia, or concomitant use of other QT-prolonging drugs (176). Gastrointestinal adverse reactions such as nausea, diarrhea, and abdominal pain are also common dose-limiting factors in long-term use (177). Therefore, when considering azithromycin for long-term management of RPI, its benefits and risks should be evaluated on an individualized basis, with close monitoring of resistance and cardiac safety. Furthermore, evidence for the immunomodulatory effects of azithromycin is mainly derived from patients with CF and children, and its effects in adult non-CF populations remain to be validated.

### 3.2 Immunomodulation and microbiome-targeted interventions

The excessive inflammatory response provoked by pathogens represents a critical mechanism that drives lung tissue injury and perpetuates the vicious cycle of infection and damage. This is particularly relevant in infantile infections, where mucosal injury can facilitate bacterial colonization and ultimately lead to irreversible consequences such as bronchiectasis (178, 179). Consequently, modulating the immune response and restoring pulmonary ecological balance have emerged as promising avenues warranting further exploration (180, 181). Animal studies have demonstrated that oral administration of Lactobacillus rhamnosus CRL1505 can activate alveolar macrophages and enhance the production of type I interferons and IFN-γ, thereby promoting clearance of RSV and alleviating lung tissue injury (181). The underlying mechanism also involves the induction of trained immunity, which confers long-term protection against subsequent pneumococcal infection in the host (182). In children, a few studies suggest that CRL1505 may reduce the incidence and severity of viral airway infections (183), but more evidence is needed to prove its efficacy. In addition, the current evidence base for probiotics in the prevention of pediatric respiratory infections remains limited, with considerable heterogeneity among studies. Their exact role in recurrent respiratory tract infections cannot yet be recommended for clinical practice and requires further investigation (184).

### 3.3 Vaccination and specific immunoprophylaxis

Adaptive immunity is the cornerstone of long-term host protection. Specific antibodies and immunological memory induced either through natural infection or vaccination confer robust defense against pathogens (185). Vaccination represents one of the most cost-effective strategies for preventing recurrent pulmonary infections, vaccines targeting common pathogens such as *Streptococcus pneumoniae* and influenza viruses have been shown to significantly reduce infection risk (185). In patients with recurrent pneumonia, booster pneumococcal vaccination has been shown in observational studies to potentially help prevent further infections (186), but validation from randomized controlled trials is still lacking. Among non-immunosuppressed patients aged 50 to 85 years hospitalized with community-acquired pneumonia, those with a four-fold or greater increase in antibody titers after vaccination had a lower risk of pneumonia recurrence in observational data than those with poorer antibody responses (187). but more data may be needed to support this result. In addition, vaccine efficacy varies across different populations. In immunocompromised individuals, the effectiveness of RSV vaccines in preventing hospitalization during the first quarter was approximately 65% to 75%, yet this protective effect waned over time (188). For children with recurrent ear, nose, and throat infections, low baseline antibody titers against pneumococcal vaccines are a common finding (186), and recurrent pneumonia has been significantly associated with a lack of response to multiple pneumococcal serotypes (189).

Not all respiratory pathogens currently have corresponding vaccines available. Mucosal vaccines targeting pathogens such as RSV, pertussis, and tuberculosis remain under investigation. These vaccines, administered via intranasal or inhalation routes, can elicit strong local immune responses at the site of infection, representing a promising area of research (190). Compared with conventional vaccines, mucosal vaccines are more effective at inducing both local and systemic immune responses (191). The uptake of licensed RSV vaccines in adult populations remains insufficient, with vaccine hesitancy, information gaps, and inconsistent vaccination guidelines being major obstacles (192). Mucosal influenza vaccines have demonstrated favorable protective efficacy in children (vaccine efficacy 62%), although the effectiveness varies by route of administration (190). The experience gained during the COVID-19 pandemic has further underscored the immense value of vaccines, with multiple technological platforms demonstrating high efficacy in preventing infection and severe disease (193). Implementing standardized vaccination may be one of the important strategies for building herd immunity and reducing infection recurrence. However, the value of adult vaccines remains underestimated (192). Future efforts should be directed toward developing effective vaccines against pathogens that currently lack vaccine coverage and optimizing vaccination strategies across different populations (185).

### 3.4 Comprehensive supportive care and public health measures

Beyond pharmacological therapy, comprehensive supportive care and broad-spectrum public health interventions play a pivotal role in improving patient outcomes and interrupting the chain of recurrence. At the clinical level, effective airway management plays an important role. Relevant measures include promoting secretion clearance through postural drainage, deep breathing exercises, and non-invasive ventilation when necessary, thereby maintaining lung function and potentially helping to reduce the retention of infectious foci (194). For patients with swallowing dysfunction, implementing strict feeding management and positional adjustments may help reduce the risk of overt or silent aspiration, thereby reducing the source of infection (195). Enhancing daily oral care may help reduce oropharyngeal colonization by pathogenic bacteria and further decrease the likelihood of pathogen migration to the lower respiratory tract (196). Comprehensive nutritional support is an important support for maintaining host immunity, and adequate intake of vitamins and minerals may help improve overall immune function (197, 198). At the broader public health level, the experience of the COVID-19 pandemic has underscored the importance of non-pharmaceutical interventions. Strengthening hand hygiene, maintaining physical distancing, and promoting respiratory etiquette in the general population can effectively interrupt transmission chains. Real-world data indicate that the implementation of such measures is associated with a reduced frequency of acute exacerbations in patients with chronic lung diseases such as bronchiectasis (199, 200). These multi-tiered and systematic strategies collectively constitute an important peripheral barrier for infection prevention and control, and may be of great significance for reducing the burden of recurrent pulmonary infection and improving patients’ long-term quality of life (201). It should be particularly noted that evidence for oral care and aspiration prevention is mainly derived from elderly patients and those with swallowing dysfunction, and their effects in other populations require further validation.

In summary, the prevention and treatment strategies for RPI encompass anti-infective therapy, immunomodulation, vaccination, and comprehensive supportive care, as summarized in **Table 1**.

**Table 1:** Prevention and treatment strategies for recurrent pulmonary infection.

Item	Specific strategies	Representative agents or measures	Level of evidence	Key evidence and limitations
Anti-infective therapy	Targeted therapy based on susceptibility	Sensitive antibiotics selected according to antimicrobial susceptibility testing results	High	Reduces selection of resistant strains. Empirical use remains common (170)
	Mucolytic adjuvant therapy	Ambroxol	Low	Increases antibiotic concentrations within lung tissue (171)
Immunomodulation	Macrolide immunomodulation	Azithromycin	Moderate	Promotes M2 macrophage polarization and attenuates inflammation (172, 174). Early-life administration reduces the risk of severe lower respiratory disease (173). Long-term resistance risk requires evaluation.
	Microbiome intervention	Probiotics (Lactobacillus rhamnosus CRL1505)	Low	Animal studies demonstrate activation of alveolar macrophages and induction of trained immunity (181, 182). In children, reduces the incidence and severity of viral airway infections (183). Strain selection and protocols are not yet standardized (184).
Vaccination	Pneumococcal vaccines	PCV and PPSV	Moderate	Booster vaccination may reduce recurrence risk (186). However, validation from randomized controlled trials is still lacking. Poor responders show limited protection (186, 189). Cross-protection between serotypes is limited.
	Influenza vaccines	Inactivated and live-attenuated vaccines	High	Suitable for elderly and chronically ill patients, with annual vaccination required to counter antigenic drift (185).
	RSV vaccines	Recombinant protein vaccines	Moderate	Effectiveness against hospitalization in immunocompromised individuals is approximately 65%–75% in the first quarter, but protection wanes over time (188). Adult uptake is affected by vaccine hesitancy (192).
	Broad-spectrum vaccines (under development)	Vaccines targeting conserved pneumococcal proteins (PspA)	Low	Hold promise for overcoming capsular serotype limitations (185) and are still in the development stage.
	Mucosal vaccines (under development)	Intranasal or inhalation-route vaccines	Low	Induce local immune responses at the site of infection (190). Efficacy in children is approximately 62% (190). Delivery technology and stability remain challenging (191), and they are still in the development stage.
Comprehensive support	Airway clearance	Postural drainage, deep breathing exercises, and non-invasive ventilation	Moderate	Facilitate secretion clearance and reduce retention of infectious foci (194).
	Aspiration prevention	Feeding management and positional adjustments	Moderate	Reduce the risk of silent aspiration in patients with swallowing dysfunction (195).
	Oral care	Professional oral cleaning	Moderate	Reduce oropharyngeal colonization by pathogens (196). In elderly patients, recurrence rate decreased from 44.7% to 27.5% (153).
	Nutritional support	Adequate intake of vitamins and minerals	Low	Fundamental for maintaining immune competence (197, 198).
	Public health measures	Hand hygiene, social distancing, and respiratory etiquette	Moderate	Have been shown to reduce acute exacerbations in patients with chronic lung diseases (199, 200).

PCV, pneumococcal conjugate vaccine; PPSV, pneumococcal polysaccharide vaccine; RSV, respiratory syncytial virus.

## 4 Recurrent pulmonary infection in low- and middle-income countries

Low- and middle-income countries (LMICs) bear the primary burden of global deaths from acute respiratory infections. Limited healthcare infrastructure, a high prevalence of malnutrition, exposure to indoor air pollution, and insufficient vaccine accessibility converge to amplify the risk of infection recurrence (202).

Epidemiological data indicate that the disease burden of RPI in LMICs is significantly higher than in high-income countries. *Streptococcus pneumoniae* is an important pathogen in pediatric recurrent pneumonia. A systematic review and meta-analysis showed that before the introduction of pneumococcal conjugate vaccine (PCV), the pooled estimate of nasopharyngeal carriage rate of *streptococcus pneumoniae* e among children under 5 years in low-income countries was as high as 64.8% (95% CI 49.8%-76.1%), and 47.8% (95% CI 44.7%-50.8%) in middle-income countries (53). Tuberculosis is also an important cause of RPI in these regions. A systematic review and meta-analysis covering 145 studies reported a pooled incidence of recurrence after tuberculosis cure of 2.26 per 100 person-years (95% CI 1.87-2.73), with approximately 70% of recurrences attributed to endogenous reactivation (203).

Insufficient diagnostic capacity is one of the core bottlenecks in RPI management in LMIC settings. Microbiological confirmation is often unavailable at primary healthcare facilities, leading to widespread empirical antibiotic use and consequently exacerbating antimicrobial resistance. In sub-Saharan Africa, antimicrobial resistance rates in childhood pneumonia continue to rise, posing severe challenges for clinical management (204). In addition, the high prevalence of immunocompromising conditions such as HIV infection and malnutrition renders patients more susceptible to opportunistic infections and recurrent episodes. In HIV-infected children, recurrent pneumonia is an important indicator of immunosuppression and requires differentiation from tuberculosis and lymphoid interstitial pneumonia (205). A mutually reinforcing vicious cycle exists between pneumonia and malnutrition, whereby recurrent infection can worsen malnutrition, and malnutrition further impairs immune function and increases the risk of reinfection (206).

For RPI prevention and treatment in LMIC settings, comprehensive strategies adapted to local conditions are required. Expanding coverage of PCV and influenza vaccines constitutes a foundational measure for reducing the incidence and recurrence of childhood pneumonia. However, sustained post-introduction surveillance is critical, as vaccine-type pneumococcal carriage may persist after vaccination (207). Hand hygiene interventions have demonstrated clear protective effects in LMICs. A systematic review and meta-analysis showed that interventions promoting handwashing with soap can significantly reduce the incidence of acute respiratory infections in LMICs (RR 0.83, 95% CI 0.76-0.90) (208). Reducing household air pollution is also of great significance. A systematic review and meta-analysis found that exposure to indoor air pollution from unprocessed solid fuels in children under 5 years was associated with an approximately 1.8-fold increased risk of pneumonia (209). Furthermore, strengthening nutritional support, improving accessibility of primary healthcare systems, and integrating RPI management into existing tuberculosis and HIV programs are all important measures suited to LMIC settings.

However, high-quality epidemiological data from LMICs remain scarce. Existing studies are mostly concentrated in single countries or regions, lacking multicenter, long-term follow-up cohort data. Future prospective studies specifically designed for LMIC settings are urgently needed to clarify the etiological composition of RPI, the evolution of resistance profiles, and effective intervention strategies, thereby providing evidence for local clinical practice and public health policy.

## 5 Conclusion and future perspectives

Recurrent pulmonary infection (RPI) is a complex clinical syndrome that fundamentally arises from the disruption of the dynamic equilibrium among pathogens, host immune responses, and pulmonary structural integrity, thereby forming a vicious cycle (**Figure 1**). This review describes the core mechanisms and key risk factors underlying RPI. The foremost challenge currently confronting this field is the blurred boundary between pathogen colonization and invasive infection. The ubiquity of asymptomatic carriage often renders etiological diagnosis difficult in terms of attribution. Building upon this, the “double-edged sword” nature of host immune responses further complicates clinical management, because the balance between protective immunity and immunopathological injury is difficult to regulate with precision. An even more challenging issue is the marked etiological heterogeneity of RPI, which encompasses primary and acquired immunodeficiencies, autoimmune diseases, drug-related effects, and local anatomical or functional abnormalities. This diversity dictates that single-dimensional interventions cannot simultaneously block the three recurrence pathways involving microbial factors, host immune status, and structural derangements.

**Figure 1:**
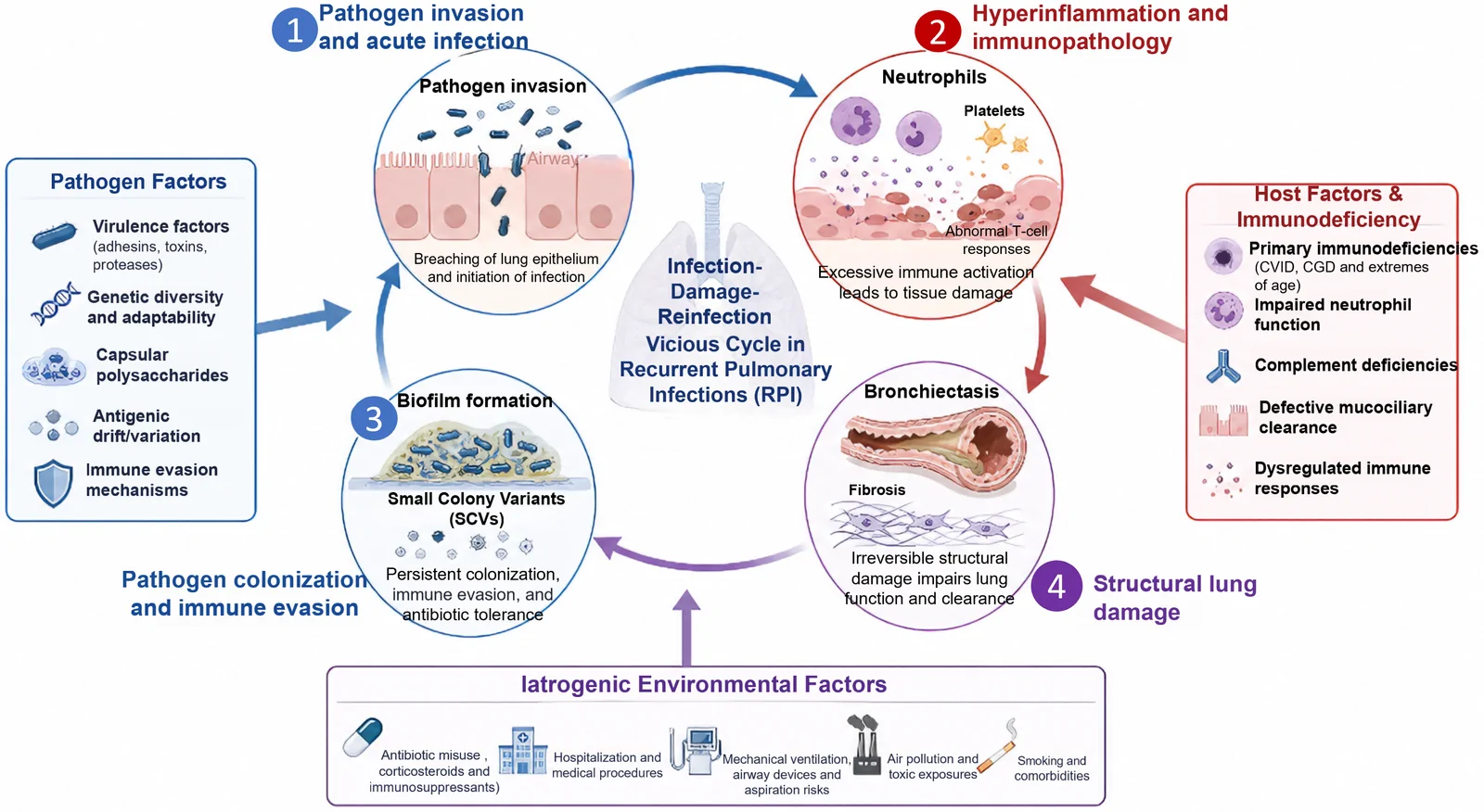
The vicious cycle of infection, damage, and reinfection in recurrent pulmonary infection.

Therefore, to overcome the bottlenecks in clinical management of RPI, future research should be strategically approached from three dimensions, namely precise identification, restoration of immune homeostasis, and reinforcement of host defense.

In the realm of precision diagnosis, metagenomic next-generation sequencing (mNGS) has shown promise in identifying pathogens in pulmonary infections. Current data indicate that its detection rate surpasses that of conventional methods (63.9% versus 27.8%), although 47.1% of positive results were reclassified as colonizing organisms after comprehensive clinical evaluation (210). A multicenter randomized controlled trial in patients with severe community-acquired pneumonia demonstrated that mNGS combined with conventional methods shortened the time to clinical improvement from 13 days to 10 days (211). However, the clinical scenario of RPI is considerably more complex. Multiple hospitalizations in patients with recurrent infections increase the likelihood of admixture between colonizers and true pathogens, and the high prevalence of asymptomatic carriage further complicates interpretation of mNGS results. For example, the carriage rate of Streptococcus pneumoniae in healthy Southeast Asian children is 36.0%, and the PCR detection rate for Mycoplasma pneumoniae in asymptomatic children is 21.2% (50, 62). At present, it remains uncertain whether mNGS can improve clinical outcomes across different types of pneumonia (community-acquired, hospital-acquired, or ventilator-associated), and relevant systematic reviews are ongoing (212). Therefore, the clinical application of mNGS in RPI should be advanced with caution. At the current stage, priority should be given to standardizing testing procedures and bioinformatic analysis, so as to avoid unnecessary antibiotic exposure resulting from overinterpretation of positive findings.

At the interventional level, modulating the respiratory or intestinal microbiota to enhance host defense represents a promising direction for exploration. A randomized double-blind placebo-controlled trial involving 120 children with recurrent respiratory tract infections demonstrated that 180 days of probiotic supplementation significantly shortened the duration and reduced the frequency of recurrence episodes for fever, cough, upper respiratory tract infections, tracheitis/bronchitis, pneumonia, and overall recurrent respiratory infections (213). Nevertheless, the evidence base for probiotics in RPI prevention and treatment remains limited, with considerable heterogeneity across studies in terms of strain selection, intervention duration, and outcome measures. Respiratory microbiome interventions are still in their nascent stages. Although potential candidate strains have been identified, their clinical translation warrants further validation through high-quality research (214). The immunomodulatory effects of azithromycin also merit attention. Studies have shown that azithromycin can promote macrophage polarization toward the M2 anti-inflammatory phenotype and reduce the release of pro-inflammatory cytokines through inhibition of the STAT1 and NF-κB signaling pathways (174). However, the balance between the risks of long-term resistance and clinical benefits requires additional evaluation. Future efforts should prioritize large-sample randomized controlled trials targeting specific RPI subtypes, so as to define the optimal strain combinations, suitable populations, and therapeutic regimens for microbiome-based interventions.

In the domain of vaccine development and application, deepening vaccination strategies represents an important avenue for reducing the disease burden of RPI. For *Streptococcus pneumoniae*, broad-spectrum vaccines targeting conserved proteins shared by all serotypes, such as PspA, are currently under development and hold promise for overcoming the limitations imposed by capsular serotype specificity (215). This direction carries particular significance for RPI, given that carriage rates of *Streptococcus pneumoniae* in children from low-income countries reach as high as 64.8%, and cross-protective immunity between different serotypes remains limited. Multi-serotype reinfection is an important immunological basis for RPI (53). The development of mucosal vaccines is equally noteworthy. Compared with intramuscular vaccines, mucosal vaccines administered via intranasal or inhalation routes can induce local immune responses at the earliest stage of pathogen invasion (216), yet they face substantial challenges including immune tolerance, precise delivery, and stability (217). Furthermore, for pathogens lacking effective vaccines, such as *Mycobacterium tuberculosis*, the protective efficacy of bacille Calmette-Guérin against adult pulmonary tuberculosis varies widely, ranging from 0% to 80%, and the widespread occurrence of exogenous reinfection in high-burden regions further limits the protection conferred by the current vaccine (83). Future work should continue to advance the development of broad-spectrum vaccines based on conserved antigens, optimize mucosal vaccine delivery technologies, and intensify clinical evaluation of novel tuberculosis vaccine candidates.

In addition, constructing an integrated comprehensive management framework is also of great significance. Combining supportive therapies such as airway clearance, nutritional support, and aspiration prevention with public health measures including hand hygiene and physical distancing can establish a holistic protection system spanning from hospital to home for patients.

In conclusion, research on RPI is transitioning from describing risk factors toward dissecting the specific molecular pathways underlying distinct etiological subtypes. The core task for the future lies in promoting a conceptual shift from passive anti-infective approaches to active host-directed therapy and integrated prevention. Through multidisciplinary collaboration, it may be possible to break the vicious cycle of infection, injury, and reinfection. If this shift can be realized, it may help reduce the disease burden of RPI and improve patients’ long-term outcomes.

## References

[1] Montella S Corcione A Santamaria F . Recurrent pneumonia in children: A reasoned diagnostic approach and a single centre experience. Int J Mol Sci. (2017) 18. doi: 10.3390/ijms18020296 28146079 PMC5343832

[2] Patria MF Esposito S . Recurrent lower respiratory tract infections in children: A practical approach to diagnosis. Paediatric Respir Rev. (2013) 14:53–60. doi: 10.1016/j.prrv.2011.11.001 23347661

[3] Boyle DP Zembower TR Qi C . Relapse versus reinfection of Mycobacterium avium complex pulmonary disease. Patient characteristics and macrolide susceptibility. Ann Am Thorac Soc. (2016) 13:1956–61. doi: 10.1513/annalsats.201605-344bc 27599038

[4] Rello J Mariscal D March F Jubert P Sanchez F Valles J . Recurrent Pseudomonas aeruginosa pneumonia in ventilated patients: relapse or reinfection? Am J Respir Crit Care Med. (1998) 157:912–6. doi: 10.1164/ajrccm.157.3.9703014 9517611

[5] Catanzaro A . Diagnosis, differentiating colonization, infection, and disease. Clinics Chest Med. (2002) 23:599–601. doi: 10.1016/s0272-5231(02)00020-5 12370995

[6] Eshwara VK Mukhopadhyay C Rello J . Community-acquired bacterial pneumonia in adults: An update. Indian J Med Res. (2020) 151:287–302. doi: 10.4103/ijmr.ijmr_1678_19 32461392 PMC7371062

[7] Quinton LJ Mizgerd JP . Dynamics of lung defense in pneumonia: resistance, resilience, and remodeling. Annu Rev Physiol. (2015) 77:407–30. doi: 10.1146/annurev-physiol-021014-071937 25148693 PMC4366440

[8] Jain S Self WH Wunderink RG Fakhran S Balk R Bramley AM . Community-acquired pneumonia requiring hospitalization among U.S. adults. N Engl J Med. (2015) 373:415–27. doi: 10.1056/nejmoa1500245 26172429 PMC4728150

[9] Sahin-Yilmaz A Naclerio RM . Anatomy and physiology of the upper airway. Proc Am Thorac Soc. (2011) 8:31–9. doi: 10.1513/pats.201007-050rn 21364219

[10] Kradin RL Digumarthy S . The pathology of pulmonary bacterial infection. Semin Diagn Pathol. (2017) 34:498–509. doi: 10.1053/j.semdp.2017.06.001 28655479

[11] Marrie TJ Lau CY Wheeler SL Wong CJ Feagan BG . Predictors of symptom resolution in patients with community-acquired pneumonia. Clin Infect Dis. (2000) 31:1362–7. doi: 10.1086/317495 11096003

[12] Mac Aogáin M Dicker AJ Mertsch P Chotirmall SH . Infection and the microbiome in bronchiectasis. Eur Respir Rev. (2024) 33:240038. doi: 10.1183/16000617.0038-2024 PMC11220623 38960615

[13] Inchingolo R Pierandrei C Montemurro G Smargiassi A Lohmeyer FM Rizzi A . Antimicrobial resistance in common respiratory pathogens of chronic bronchiectasis patients: A literature review. Antibiotics. (2021) 10:326. doi: 10.3390/antibiotics10030326 33804631 PMC8003644

[14] Chalmers JD Chang AB Chotirmall SH Dhar R McShane PJ . Bronchiectasis. Nat Rev Dis Primers. (2018) 4:45. doi: 10.1513/annalsats.201710-832ed 30442957

[15] GBD 2017 Influenza Collaborators . Mortality, morbidity, and hospitalisations due to influenza lower respiratory tract infections, 2017: an analysis for the Global Burden of Disease Study 2017. Lancet Respir Med. (2019) 7:69–89. doi: 10.1016/S2213-2600(18)30496-X PMC6302221 30553848

[16] Perin J Mulick A Yeung D Villavicencio F Lopez G Strong KL . Global, regional, and national causes of under-5 mortality in 2000-19: an updated systematic analysis with implications for the Sustainable Development Goals. Lancet Child Adolesc Health. (2022) 6:106–15. doi: 10.1016/s2352-4642(21)00311-4 34800370 PMC8786667

[17] Edmond K Scott S Korczak V Ward C Sanderson C Theodoratou E . Long term sequelae from childhood pneumonia; systematic review and meta-analysis. PloS One. (2012) 7:e31239. doi: 10.1371/journal.pone.0031239 22384005 PMC3285155

[18] Johnston ID . Effect of pneumonia in childhood on adult lung function. J Pediatr. (1999) 135:33–7. 10431137

[19] Mayr FB Yende S Angus DC . Epidemiology of severe sepsis. Virulence. (2014) 5:4–11. doi: 10.4161/viru.27372 PMC3916382 24335434

[20] Calfee CS Janz DR Bernard GR May AK Kangelaris KN Matthay MA . Distinct molecular phenotypes of direct vs indirect ARDS in single-center and multicenter studies. Chest. (2015) 147:1539–48. doi: 10.1378/chest.14-2454 26033126 PMC4451708

[21] Özçelik T Can Oksay S Girit S . Diagnostic approach to the etiology of recurrent pneumonia in children. Pediatr Pulmonology. (2024) 59:3650–9. doi: 10.1002/ppul.27275 PMC11601008 39291831

[22] Tang Q Chen X Ran K Yang X Yuan T Li J . Advances in understanding recurrent pulmonary infections following foreign body aspiration: A narrative review. J Asthma Allergy. (2025) 18:1093–103. doi: 10.2147/jaa.s524781 40689224 PMC12275911

[23] Hoving MFP Brand PLP . Causes of recurrent pneumonia in children in a general hospital. J Paediatrics Child Health. (2013) 49:E208–12. doi: 10.1111/jpc.12114 23438187

[24] Dang TT Majumdar SR Marrie TJ Eurich DT . Recurrent pneumonia: A review with focus on clinical epidemiology and modifiable risk factors in elderly patients. Drugs Aging. (2014) 32:13–9. doi: 10.1007/s40266-014-0229-6 25491559

[25] Ekdahl K Braconier JH Rollof J . Recurrent pneumonia: A review of 90 adult patients. Scandinavian J Infect Dis. (2009) 24:71–6. doi: 10.3109/00365549209048403 1589728

[26] Takada K Ogawa K Miyamoto A Nakahama H Moriguchi S Murase K . Risk factors and interventions for developing recurrent pneumonia in older adults. ERJ Open Res. (2023) 9:00516–2022. doi: 10.1183/23120541.00516-2022 37143835 PMC10152262

[27] Meyer KC . Lung infections and aging. Ageing Res Rev. (2004) 3:55–67. doi: 10.1016/j.arr.2003.07.002 15163102 PMC7129100

[28] Meyer K . The role of immunity and inflammation in lung senescence and susceptibility to infection in the elderly. Semin Respir Crit Care Med. (2010) 31:561–74. doi: 10.1055/s-0030-1265897 20941657

[29] Høiby N . Cystic fibrosis and the clinical biofilm revolution a survey of the Danish CF Center's contribution. Biofilm. (2025) 9. doi: 10.1016/j.bioflm.2024.100246 PMC11732244 39811797

[30] Ribeiro CMP Higgs MG Muhlebach MS Wolfgang MC Borgatti M Lampronti I . Revisiting host-pathogen interactions in cystic fibrosis lungs in the era of CFTR modulators. Int J Mol Sci. (2023) 24. doi: 10.3390/ijms24055010 36902441 PMC10003689

[31] Fu Z Sun Y Yao H Liu Q Zhang Q Hu J . A diagnostic model based on pulmonary microbiota and host gene expression to distinguish colonization from pneumonia. Sci Rep. (2026) 16. doi: 10.1038/s41598-026-44972-w 41933095 PMC13194893

[32] Garcia-Vidal C Carratalà J Fernández-Sabé N Dorca J Verdaguer R Manresa F . Aetiology of, and risk factors for, recurrent community-acquired pneumonia. Clin Microbiol Infection. (2009) 15:1033–8. doi: 10.1111/j.1469-0691.2009.02918.x 19673961

[33] Clementi N Ghosh S De Santis M Castelli M Criscuolo E Zanoni I . Viral respiratory pathogens and lung injury. Clin Microbiol Rev. (2021) 34. doi: 10.1128/cmr.00103-20 33789928 PMC8142519

[34] Deshpande R Zou C . Pseudomonas aeruginosa induced cell death in acute lung injury and acute respiratory distress syndrome. Int J Mol Sci. (2020) 21. doi: 10.3390/ijms21155356 32731491 PMC7432812

[35] Dang TT Majumdar SR Marrie TJ Eurich DT . Recurrent pneumonia: a review with focus on clinical epidemiology and modifiable risk factors in elderly patients. Drugs Aging. (2015) 32:13–9. doi: 10.1007/s40266-014-0229-6 25491559

[36] Verhagen LM de Groot R . Recurrent, protracted and persistent lower respiratory tract infection: A neglected clinical entity. J Infect. (2015) 71:S106–11. doi: 10.1016/j.jinf.2015.04.011 25917807

[37] Mena-Vázquez N Redondo-Rodriguez R Rojas-Gimenez M Romero-Barco CM Fuego-Varela C Perez-Gómez N . Rate of severe and fatal infections in a cohort of patients with interstitial lung disease associated with rheumatoid arthritis: a multicenter prospective study. Front Immunol. (2024) 15:1341321. doi: 10.3389/fimmu.2024.1341321 PMC11007097 38605950

[38] Adivitiya Kaushik MS Chakraborty S Veleri S Kateriya S . Mucociliary respiratory epithelium integrity in molecular defense and susceptibility to pulmonary viral infections. Biology. (2021) 10. doi: 10.3390/biology10020095 33572760 PMC7911113

[39] Yum H-K Park IN Shin B-M Choi S-J . Recurrent Pseudomonas aeruginosa infection in chronic lung diseases: relapse or reinfection? Tuberculosis Respir Dis. (2014) 77. doi: 10.4046/trd.2014.77.4.172 PMC4217033 25368663

[40] Rastegar-Kashkouli A Khaledi A Moammer F Ghenaatpisheh Sanani M Heidari MM Foroughi Eghbal A . Systematic review and meta-analysis on the prevalence and antibiotic susceptibility pattern in Pseudomonas aeruginosa isolated from cystic fibrosis patients. Eurasian J Med. (2024) 56:189–98. doi: 10.5152/eurasianjmed.2024.23302 39655837 PMC11535318

[41] Tural-Kara T Özdemir H Yıldız N Aldemir Kocabaş B Erat T Yahşi A . Underlying diseases and causative microorganisms of recurrent pneumonia in children: A 13-year study in a university hospital. J Trop Pediatr. (2019) 65:224–30. doi: 10.1093/tropej/fmy037 PMC7107262 30011014

[42] Shoar S Musher DM . Etiology of community-acquired pneumonia in adults: a systematic review. Pneumonia. (2020) 12. doi: 10.1186/s41479-020-00074-3 33024653 PMC7533148

[43] Dale KD Globan M Tay EL Trauer JM Trevan PG Denholm JT . Recurrence of tuberculosis in a low-incidence setting without directly observed treatment: Victoria, Australia, 2002–2014. Int J Tuberculosis Lung Dis. (2017) 21:550–5. doi: 10.5588/ijtld.16.0651 28399970

[44] Marx FM Dunbar R Enarson DA Williams BG Warren RM Spuy G . The temporal dynamics of relapse and reinfection tuberculosis after successful treatment: a retrospective cohort study. Clin Infect Dis. (2014) 58:1676–83. doi: 10.1093/cid/ciu186 24647020

[45] Pradhan A Ma Q de Assis LJ Leaves I Larcombe DE Rodriguez Rondon AV . Anticipatory stress responses and immune evasion in fungal pathogens. Trends Microbiol. (2021) 29:416–27. doi: 10.1016/j.tim.2020.09.010 33059975

[46] Xu J Seifert HS . Analysis of pilin antigenic variation in Neisseria meningitidis by next-generation sequencing. J Bacteriol. (2018) 200:e00465-18. doi: 10.1128/jb.00465-18 30181126 PMC6199478

[47] Rather MA Gupta K Mandal M . Microbial biofilm: formation, architecture, antibiotic resistance, and control strategies. Braz J Microbiol. (2021) 52:1701–18. doi: 10.1007/s42770-021-00624-x 34558029 PMC8578483

[48] de Jong R Tenbrock K Ohl K . New insights in immunometabolism in neonatal monocytes and macrophages in health and disease. Int J Mol Sci. (2023) 24. doi: 10.3390/ijms241814173 37762476 PMC10531550

[49] Yerolemou N Richardson NI Ravenscroft N Kuttel MM . Molecular modeling of Streptococcus pneumoniae serogroup 23 capsular polysaccharide conformations provides insight into antibody binding and cross-reactivity. Carbohydr Res. (2026) 564. doi: 10.1016/j.carres.2026.109882 41812523

[50] Daningrat WOD Amalia H Ayu IM Satzke C Safari D . Carriage of Streptococcus pneumoniae in children under five years of age prior to pneumococcal vaccine introduction in Southeast Asia: A systematic review and meta-analysis (2001–2019). J Microbiol Immunol Infect. (2022) 55:6–17. doi: 10.1016/j.jmii.2021.08.002 34511388

[51] Trotter CL Usuf E Bottomley C Adegbola RA Hall A . Pneumococcal carriage in sub-Saharan Africa—a systematic review. PloS One. (2014) 9. doi: 10.1371/journal.pone.0085001 PMC3896352 24465464

[52] Darboe MK Fulford AJC Secka O Prentice AM . The dynamics of nasopharyngeal streptococcus pneumoniae carriage among rural Gambian mother-infant pairs. BMC Infect Dis. (2010) 10. doi: 10.1186/1471-2334-10-195 20602782 PMC2910019

[53] Reid SD Adegbola RA DeAntonio R Hill PC Roca A Usuf E . Carriage of Streptococcus pneumoniae and other respiratory bacterial pathogens in low and lower-middle income countries: a systematic review and meta-analysis. PloS One. (2014) 9. doi: 10.1371/journal.pone.0103293 PMC4118866 25084351

[54] Mcdaniel LS Sheffield JS Delucchi P Briles DE . PspA, a surface protein of Streptococcus pneumoniae, is capable of eliciting protection against pneumococci of more than one capsular type. (1991) 59:222–8. 10.1128/iai.59.1.222-228.1991 PMC257730 1987036

[55] Malley R Srivastava A Lipsitch M Thompson CM Watkins C Tzianabos A . Antibody-independent, interleukin-17A-mediated, cross-serotype immunity to pneumococci in mice immunized intranasally with the cell wall polysaccharide. Infect Immun. (2006) 74:2187–95. doi: 10.1128/iai.74.4.2187-2195.2006 16552049 PMC1418935

[56] Wang Y Jiang B Guo Y Li W Tian Y Sonnenberg GF . Cross-protective mucosal immunity mediated by memory Th17 cells against Streptococcus pneumoniae lung infection. Mucosal Immunol. (2017) 10:250–9. doi: 10.1038/mi.2016.41 27118490 PMC5083242

[57] Feemster K Hausdorff WP Banniettis N Platt H Velentgas P Esteves-Jaramillo A . Implications of cross-reactivity and cross-protection for pneumococcal vaccine development. Vaccines. (2024) 12. doi: 10.3390/vaccines12090974 39340006 PMC11435891

[58] Ma C Zhang Y Wang H . Characteristics of Haemophilus influenzae carriage among healthy children in China: a meta-analysis. Medicine. (2023) 102. doi: 10.1097/md.0000000000035313 37933036 PMC10627696

[59] Yang P Zhang J Peng A . The pharyngeal carriage of Haemophilus influenzae among healthy population in China: a systematic review and meta-analysis. BMC Infect Dis. (2019) 19. doi: 10.1186/s12879-019-4195-9 31226950 PMC6588945

[60] De Schutter I De Wachter E Crokaert F Verhaegen J Soetens O Pierard D . Microbiology of bronchoalveolar lavage fluid in children with acute nonresponding or recurrent community-acquired pneumonia: identification of nontypeable Haemophilus influenzae as a major pathogen. Clin Infect Dis. (2011) 52:1437–44. doi: 10.1093/cid/cir235 21628484 PMC7107807

[61] King PT Sharma R . The lung immune response to nontypeableHaemophilus influenzae(lung immunity to NTHi). J Immunol Res. (2015) 2015:1–14. doi: 10.1155/2015/706376 26114124 PMC4465770

[62] Klugman KP Spuesens EBM Fraaij PLA Visser EG Hoogenboezem T Hop WCJ . Carriage of Mycoplasma pneumoniae in the upper respiratory tract of symptomatic and asymptomatic children: an observational study. PloS Med. (2013) 10. doi: 10.1371/journal.pmed.1001444 PMC3653782 23690754

[63] Koenen MH de Groot RCA de Steenhuijsen Piters WAA Chu MLJN Arp K Hasrat R . Mycoplasma pneumoniae carriage in children with recurrent respiratory tract infections is associated with a less diverse and altered microbiota. eBioMedicine. (2023) 98. doi: 10.1016/j.ebiom.2023.104868 37950996 PMC10679896

[64] Cole AL Marzahn MR Valdespino EL Patel R Cole AM . Nasal microbiome inhabitants with anti-Staphylococcus aureus activity. Microbiol Spectr. (2025) 13:e0102425. doi: 10.1128/spectrum.01024-25 PMC12502565 40928460

[65] Pivard M Moreau K Vandenesch F Fey PD . Staphylococcus aureus arsenal to conquer the lower respiratory tract. mSphere. (2021) 6. doi: 10.1128/msphere.00059-21 34011681 PMC8265621

[66] Morris AJ Eisha S Yau YCW DePas WH Fabiano G Aberdein S . The effect of Staphylococcus aureus on the persistence of Pseudomonas aeruginosa due to aggregation in cystic fibrosis airway infections. iScience. (2026) 29. doi: 10.1016/j.isci.2026.116016 42325576 PMC13276413

[67] Aliberti S Reyes LF Faverio P Sotgiu G Dore S Rodriguez AH . Global initiative for meticillin-resistant Staphylococcus aureus pneumonia (GLIMP): an international, observational cohort study. Lancet Infect Dis. (2016) 16:1364–76. doi: 10.1016/s1473-3099(16)30267-5 27593581

[68] Xu H Wang S Liu X Li M Wang X Chen H . Strategies for survival of Staphylococcus aureus in host cells. Int J Mol Sci. (2025) 26. doi: 10.3390/ijms26020720 39859434 PMC11765632

[69] Howden BP Giulieri SG Wong Fok Lung T Baines SL Sharkey LK Lee JYH . Staphylococcus aureus host interactions and adaptation. Nat Rev Microbiol. (2023) 21:380–95. doi: 10.1038/s41579-023-00852-y 36707725 PMC9882747

[70] Kahl BC . Small colony variants (SCVs) of Staphylococcus aureus – a bacterial survival strategy. Infection Genet Evol. (2014) 21:515–22. doi: 10.1016/j.meegid.2013.05.016 23722021

[71] Eifr C Proctor RA Peters G . Small colony variants of Staphylococci: a link to persistent infections. Berliner und Munchener tierarztliche Wochenschrift. (2000) 113:321–5. 11042943

[72] Principi N Esposito S . Biofilm production and its implications in pediatrics. Microorganisms. (2024) 12. doi: 10.3390/microorganisms12081522 39203365 PMC11356046

[73] Liu Y Guo S Hu X Guo Q Lu F . Community-acquired pneumonia with Staphylococcus aureus and viral co-infection: clinical characteristics and pathogen genomic analysis. J Thorac Dis. (2026) 18:338. doi: 10.21037/jtd-2025-1-2791 42182636 PMC13190221

[74] Wardenburg JB Bae T Otto M DeLeo4 FR Schneewind1 O . Poring over pores: alpha-hemolysin and Panton-Valentine leukocidin in Staphylococcus aureus pneumonia. Nat Med. (2007) 13:1405–6. doi: 10.1038/nm1207-1405 18064027

[75] Sindeldecker D Stoodley P . The many antibiotic resistance and tolerance strategies of Pseudomonas aeruginosa. Biofilm. (2021) 3:100056. doi: 10.1016/j.bioflm.2021.100056 34471871 PMC8387898

[76] Maurice NM Bedi B Sadikot RT . Pseudomonas aeruginosa biofilms: host response and clinical implications in lung infections. Am J Respir Cell Mol Biol. (2018) 58:428–39. doi: 10.1165/rcmb.2017-0321TR PMC5894500 29372812

[77] Kunz Coyne AJ El Ghali A Holger D Rebold N Rybak MJ . Therapeutic strategies for emerging multidrug-resistant Pseudomonas aeruginosa. Infect Dis Ther. (2022) 11:661–82. doi: 10.1007/s40121-022-00591-2 35150435 PMC8960490

[78] Rosser A Marx FM Pareek M . Recurrent tuberculosis in the pre-elimination era. Int J Tuberc Lung Dis. (2018) 22:139–50. doi: 10.5588/ijtld.17.0590 29506610

[79] Vega V Cabrera-Sanchez J Rodríguez S Verdonck K Seas C Otero L . Risk factors for pulmonary tuberculosis recurrence, relapse and reinfection: a systematic review and meta-analysis. BMJ Open Respir Res. (2024) 11. doi: 10.1136/bmjresp-2023-002281 38479821 PMC10941165

[80] Lyon SM Rossman MD . Pulmonary tuberculosis. Microbiol Spectr. (2017) 5. doi: 10.1128/9781555819866.ch16 PMC11687454 28185620

[81] Lawrence A . Bacillus Calmette-Guérin (BCG) revaccination and protection against tuberculosis: a systematic review. Cureus (2024) 16(3):e56643. doi: 10.7759/cureus.56643 38646352 PMC11032142

[82] Cardona P-J . Reinfection as a central constraint on tuberculosis vaccine development. Front Immunol. (2026) 17:1807344. doi: 10.3389/fimmu.2026.1807344 42093996 PMC13139096

[83] Messina NL Netea MG Curtis N . The impact of human single nucleotide polymorphisms on Bacillus Calmette-Guérin responses. Vaccine. (2020) 38:6224–35. doi: 10.1016/j.vaccine.2020.07.032 32826104

[84] Wotale TW Lelisho ME Negasa BW Tareke SA Gobena WE Amesa EG . Identifying risk factors for recurrent multidrug resistant tuberculosis based on patient’s record data from 2016 to 2021: retrospective study. Sci Rep. (2024) 14. doi: 10.1038/s41598-024-73209-x 39397064 PMC11471762

[85] He W Tan Y Song Z Liu B Wang Y He P . Endogenous relapse and exogenous reinfection in recurrent pulmonary tuberculosis: a retrospective study revealed by whole genome sequencing. Front Microbiol. (2023) 14. doi: 10.3389/fmicb.2023.1115295 36876077 PMC9981662

[86] Tognarelli EI Bueno SM González PA . Immune-modulation by the human respiratory syncytial virus: focus on dendritic cells. Front Immunol. (2019) 10. doi: 10.3389/fimmu.2019.00810 31057543 PMC6478035

[87] Roberts NJ . Respiratory syncytial virus suppression of the antiviral immune response: implications for evaluation of candidate vaccines. Vaccine. (2019) 37:7451–4. doi: 10.1016/j.vaccine.2019.09.096 31607601

[88] Salkind AR Jr NJR . Recent observations regarding the pathogenesis of recurrent respiratory syncytial virus infections: implications for vaccine development. Vaccine. (1992) 10:519–23. doi: 10.1016/0264-410x(92)90350-s 1621415

[89] Maurer DP Vu M Schmidt AG . Antigenic drift expands influenza viral escape pathways from recalled humoral immunity. Immunity. (2025) 58:716–727.e6. doi: 10.1016/j.immuni.2025.02.006 40023162 PMC11906258

[90] González AE Lay MK Jara EL Espinoza JA Gómez RS Soto J . Aberrant T cell immunity triggered by human respiratory syncytial virus and human metapneumovirus infection. Virulence. (2016) 8:685–704. doi: 10.1080/21505594.2016.1265725 PMC5626346 27911218

[91] Garcia-Garcia ML Calvo Rey C Del Rosal Rabes T . Pediatric asthma and viral infection. Arch Bronconeumol. (2016) 52:269–73. doi: 10.1016/j.arbres.2015.11.008 PMC7131251 26766408

[92] Vandini S Biagi C Fischer M Lanari M . Impact of rhinovirus infections in children. Viruses. (2019) 11. doi: 10.3390/v11060521 31195744 PMC6632063

[93] Rajput C Han M Ishikawa T Lei J Jazaeri S Bentley JK . Early-life heterologous rhinovirus infections induce an exaggerated asthma-like phenotype. J Allergy Clin Immunol. (2020) 146:571–582.e3. doi: 10.1016/j.jaci.2020.03.039 32344055 PMC8097639

[94] Li Y Han M Singh S Breckenridge HA Kreger JE Stroupe CC . Tuft cells are required for a rhinovirus-induced asthma phenotype in immature mice. JCI Insight. (2024) 9. doi: 10.1172/jci.insight.166136 38061015 PMC10906234

[95] Sajjan U Wang Q Zhao Y Gruenert DC Hershenson MB . Rhinovirus disrupts the barrier function of polarized airway epithelial cells. Am J Respir Crit Care Med. (2008) 178:1271–81. doi: 10.1164/rccm.200801-136oc 18787220 PMC2599868

[96] Nawroth J Lucchesi C Cheng D Shukla A Ngyuen J Shroff T . A microengineered airway lung chip models key features of viral-induced exacerbation of asthma. Am J Respir Cell Mol Biol. (2020) 63:591–600. doi: 10.1165/rcmb.2020-0010ma 32706623

[97] Boland H Endres A Kinscherf R Schubert R Wilhelm B Schwarzbach H . Protective effect of interferon type I on barrier function of human airway epithelium during rhinovirus infections *in vitro*. Sci Rep. (2024) 14. doi: 10.1038/s41598-024-82516-2 39681656 PMC11649702

[98] Most ZM Perl TM Sebert M . Respiratory virus infections in symptomatic and asymptomatic children upon hospital admission: new insights. Antimicrob Stewardship Healthcare Epidemiol. (2024) 4. doi: 10.1017/ash.2024.407 39411664 PMC11474761

[99] Schildgen O Moe N Pedersen B Nordbø SA Skanke LH Krokstad S . Respiratory virus detection and clinical diagnosis in children attending day care. PloS One. (2016) 11. doi: 10.1371/journal.pone.0159196 PMC4951077 27433803

[100] Jansen RR Wieringa J Koekkoek SM Visser CE Pajkrt D Molenkamp R . Frequent detection of respiratory viruses without symptoms: toward defining clinically relevant cutoff values. J Clin Microbiol. (2011) 49:2631–6. doi: 10.1128/jcm.02094-10 21543571 PMC3147826

[101] Howard LM Zhu Y Griffin MR Edwards KM Williams JV Gil AI . Nasopharyngeal pneumococcal density during asymptomatic respiratory virus infection and risk for subsequent acute respiratory illness. Emerging Infect Dis. (2019) 25:2040–7. doi: 10.3201/eid2511.190157 31625844 PMC6810199

[102] Ochani RK Asad A Yasmin F Shaikh S Khalid H Batra S . COVID-19 pandemic: from origins to outcomes. A comprehensive review of viral pathogenesis, clinical manifestations, diagnostic evaluation, and management. Infezioni Med. (2021) 29:20–36. 33664170

[103] Jain VK Iyengar K Garg R Vaishya R . Elucidating reasons of COVID-19 re-infection and its management strategies. Diabetes Metab Syndrome: Clin Res Rev. (2021) 15:1001–6. doi: 10.1016/j.dsx.2021.05.008 33989898 PMC8102074

[104] Negi N Maurya SP Singh R Das BK . An update on host immunity correlates and prospects of re-infection in COVID-19. Int Rev Immunol. (2022) 41:367–92. doi: 10.1080/08830185.2021.2019727 PMC8787841 34961403

[105] Richard M Fouchier RA . Influenza A virus transmission via respiratory aerosols or droplets as it relates to pandemic potential. FEMS Microbiol Rev. (2016) 40:68–85. doi: 10.1093/femsre/fuv039 PMC5006288 26385895

[106] Petrova VN Russell CA . The evolution of seasonal influenza viruses. Nat Rev Microbiol. (2018) 16:47–60. doi: 10.1038/nrmicro.2017.118 29081496

[107] Jensen JU Hein L Lundgren B Bestle MH Mohr T Andersen MH . Invasive Candida infections and the harm from antibacterial drugs in critically ill patients: data from a randomized, controlled trial to determine the role of ciprofloxacin, piperacillin-tazobactam, meropenem, and cefuroxime. Crit Care Med. (2015) 43:594–602. doi: 10.1097/ccm.0000000000000746 25493970

[108] Huang L Li S Jiang R Lei S Wu J Huang L . Glucocorticoid use and parenteral nutrition are risk factors for catheter-related Candida bloodstream infection: a retrospective study. Asian BioMed (Res Rev News). (2024) 18:109–15. doi: 10.2478/abm-2024-0016 39175949 PMC11337845

[109] Giles SS Dagenais TR Botts MR Keller NP Hull CM . Elucidating the pathogenesis of spores from the human fungal pathogen Cryptococcus neoformans. Infect Immun. (2009) 77:3491–500. doi: 10.1128/IAI.00334-09 PMC2715683 19451235

[110] Setianingrum F Rautemaa-Richardson R Denning DW . Pulmonary cryptococcosis: a review of pathobiology and clinical aspects. Med Mycol. (2019) 57:133–50. doi: 10.1093/mmy/myy086 30329097

[111] Henriet SS Verweij PE Warris A . Aspergillus nidulans and chronic granulomatous disease: a unique host-pathogen interaction. J Infect Dis. (2012) 206:1128–37. doi: 10.1093/infdis/jis473 22829648

[112] Salvator H Mahlaoui N Catherinot E Rivaud E Pilmis B Borie R . Pulmonary manifestations in adult patients with chronic granulomatous disease. Eur Respir J. (2015) 45:1613–23. doi: 10.1183/09031936.00118414 25614174

[113] Hanada S Pirzadeh M Carver KY Deng JC . Respiratory viral infection-induced microbiome alterations and secondary bacterial pneumonia. Front Immunol. (2018) 9:2640. doi: 10.3389/fimmu.2018.02640 30505304 PMC6250824

[114] Peteranderl C Herold S Schmoldt C . Human influenza virus infections. Semin Respir Crit Care Med. (2016) 37:487–500. doi: 10.1055/s-0036-1584801 PMC7174870 27486731

[115] Kalil AC Thomas PG . Influenza virus-related critical illness: pathophysiology and epidemiology. Crit Care. (2019) 23:258. doi: 10.1186/s13054-019-2539-x 31324202 PMC6642581

[116] van Houten CB Cohen A Engelhard D Hays JP Karlsson R Moore E . Antibiotic misuse in respiratory tract infections in children and adults-a prospective, multicentre study (TAILORED Treatment). Eur J Clin Microbiol Infect Dis. (2019) 38:505–14. doi: 10.1007/s10096-018-03454-2 30707378 PMC6394715

[117] Dadashi M Khaleghnejad S Abedi Elkhichi P Goudarzi M Goudarzi H Taghavi A . COVID-19 and influenza co-infection: a systematic review and meta-analysis. Front Med (Lausanne). (2021) 8:681469. doi: 10.3389/fmed.2021.681469 34249971 PMC8267808

[118] Liang J Wang Y Lin Z He W Sun J Li Q . Influenza and COVID-19 co-infection and vaccine effectiveness against severe cases: a mathematical modeling study. Front Cell Infect Microbiol. (2024) 14:1347710. doi: 10.3389/fcimb.2024.1347710 38500506 PMC10945002

[119] Rezaee D Bakhtiari S Jalilian FA Doosti-Irani A Asadi FT Ansari N . Coinfection with severe acute respiratory syndrome coronavirus 2 (SARS-CoV-2) and influenza virus during the COVID-19 pandemic. Arch Virol. (2023) 168:53. doi: 10.1007/s00705-022-05628-y 36609722 PMC9825093

[120] Ahmed AO Ali GA Goravey W . Concomitant pulmonary tuberculosis and invasive aspergillosis infection in an immunocompetent host. Eur J Case Rep Intern Med. (2022) 9:003249. doi: 10.12890/2022_003249 PMC8988504 35402331

[121] Matthay MA Zemans RL . The acute respiratory distress syndrome: pathogenesis and treatment. Annu Rev Pathol. (2011) 6:147–63. doi: 10.1146/annurev-pathol-011110-130158 20936936 PMC3108259

[122] Josset L Menachery VD Gralinski LE Agnihothram S Sova P Carter VS . Cell host response to infection with novel human coronavirus EMC predicts potential antivirals and important differences with SARS coronavirus. mBio. (2013) 4:e00165-13. doi: 10.1128/mbio.00165-13 23631916 PMC3663187

[123] Newton AH Cardani A Braciale TJ . The host immune response in respiratory virus infection: balancing virus clearance and immunopathology. Semin Immunopathol. (2016) 38:471–82. doi: 10.1007/s00281-016-0558-0 26965109 PMC4896975

[124] Kumar V . Pulmonary innate immune response determines the outcome of inflammation during pneumonia and sepsis-associated acute lung injury. Front Immunol. (2020) 11:1722. doi: 10.3389/fimmu.2020.01722 32849610 PMC7417316

[125] Dharmarajan K Hsieh AF Lin Z Bueno H Ross JS Horwitz LI . Diagnoses and timing of 30-day readmissions after hospitalization for heart failure, acute myocardial infarction, or pneumonia. Jama. (2013) 309. doi: 10.1001/jama.2012.216476 23340637 PMC3688083

[126] Quinton LJ Walkey AJ Mizgerd JP . Integrative physiology of pneumonia. Physiol Rev. (2018) 98:1417–64. doi: 10.1152/physrev.00032.2017 29767563 PMC6088146

[127] Ben-Meir E Teper A Strug L Ratjen F Kerem E . Post-infectious bronchiolitis obliterans in children: pathogenesis, treatment strategies, and future directions. Curr Opin Pediatr. (2026) 38:454–60. doi: 10.1097/mop.0000000000001585 42234623

[128] Liu C Yuan X . Respiratory viruses as key drivers of pulmonary fibrosis: integrated pathways from barrier injury to immune–fibrotic crosstalk. Virology. (2026) 620. doi: 10.1016/j.virol.2026.110913 41996892

[129] Flume PA Chalmers JD Olivier KN . Advances in bronchiectasis: endotyping, genetics, microbiome, and disease heterogeneity. Lancet. (2018) 392:880–91. doi: 10.1016/s0140-6736(18)31767-7 30215383 PMC6173801

[130] Ren C Yao RQ Ren D Li Y Feng YW Yao YM . Comparison of clinical laboratory tests between bacterial sepsis and SARS-CoV-2-associated viral sepsis. Mil Med Res. (2020) 7:36. doi: 10.1186/s40779-020-00267-3 32753048 PMC7399032

[131] Wu M Zou ZY Chen YH Wang CL Feng YW Liu ZF . Severe COVID-19-associated sepsis is different from classical sepsis induced by pulmonary infection with carbapenem-resistant klebsiella pneumonia (CrKP). Chin J Traumatol. (2022) 25:17–24. doi: 10.1016/j.cjtee.2021.11.001 34815141 PMC8591816

[132] Gürtler G Schramm W Seitz R . Viral sepsis – pathophysiology and disease manifestation. Infection. (2025) 53:775–84. doi: 10.1007/s15010-025-02486-z PMC12137479 39961996

[133] He W Xiao K Xu J Guan W Xie S Wang K . Recurrent sepsis exacerbates CD4+ T cell exhaustion and decreases antiviral immune responses. Front Immunol. (2021) 12. doi: 10.3389/fimmu.2021.627435 33717146 PMC7946831

[134] Condotta SA Khan SH Rai D Griffith TS Badovinac VP . Polymicrobial sepsis increases susceptibility to chronic viral infection and exacerbates CD8+ T cell exhaustion. J Immunol. (2015) 195:116–25. doi: 10.4049/jimmunol.1402473 25980007 PMC4475506

[135] Choi YJ Kim SB Kim JH Park S-H Park MS Kim JM . Impaired polyfunctionality of CD8+ T cells in severe sepsis patients with human cytomegalovirus reactivation. Exp Mol Med. (2017) 49:e382-e. doi: 10.1038/emm.2017.146 28960213 PMC5628278

[136] Walton AH Muenzer JT Rasche D Boomer JS Sato B Brownstein BH . Reactivation of multiple viruses in patients with sepsis. PloS One. (2014) 9:e98819. doi: 10.1371/journal.pone.0098819 24919177 PMC4053360

[137] de Brabander J Ong DSY Cremer OL Bos LDJ Peters-Sengers H van der Poll T . Cytomegalovirus reactivation and the host immune response in patients with sepsis. J Infection. (2025) 91. doi: 10.1016/j.jinf.2025.106657 41241236

[138] Wang H Anthony D Selemidis S Vlahos R Bozinovski S . Resolving viral-induced secondary bacterial infection in COPD: a concise review. Front Immunol. (2018) 9:2345. doi: 10.3389/fimmu.2018.02345 30459754 PMC6232692

[139] Hatayama Y Hashimoto Y Motokura T . Frequent co-reactivation of Epstein–Barr virus in patients with cytomegalovirus viremia under immunosuppressive therapy and/or chemotherapy. J Int Med Res. (2020) 48. doi: 10.1177/0300060520972880 33249964 PMC7708703

[140] Özkoç S Delibas SB . Investigation of Pneumocystis jirovecii pneumonia and colonization in iatrogenically immunosuppressed and immunocompetent patients. Mikrobiyoloji Bulteni. (2015) 49:221–30. doi: 10.5578/mb.9344 26167822

[141] Kim L Ferraz C Corbisiero MF Gorvetzian S Franco‐Paredes C Krsak M . Glucocorticoids as a risk factor for infection and adverse outcomes in non‐HIV and non‐transplant patients with cryptococcal meningitis. Mycoses. (2024) 67. doi: 10.1111/myc.13709 38429225

[142] Qian Y Zhang Y Huang J Liu J Chen G Xia G . Risk factors for Pneumocystis jirovecii pneumonia in children with systemic lupus erythematosus exposed to prolonged high-dose glucocorticoids. JCR: J Clin Rheumatol. (2024) 30:e86–92. doi: 10.1097/rhu.0000000000002073 38389131

[143] Kumar V Tang Q Tang X Yin W Li Y Zhong X . Infective pneumonia following the use of tumor necrosis factor-α inhibitors in inflammatory bowel disease patients: a real-world disproportionality analysis of the FDA Adverse Event Reporting System (FAERS) database. PloS One. (2025) 20. doi: 10.1371/journal.pone.0317242 PMC12324135 40763141

[144] Li S Zhang W Ma H Gao X Yuan W He M . Prior culture-guided prediction of antibiotic susceptibility in recurrent respiratory tract infections: a retrospective cohort analysis. Front Cell Infect Microbiol. (2026) 15. doi: 10.3389/fcimb.2025.1715986 41568003 PMC12816384

[145] Kiem S Schentag JJ . Correlations between microbiological outcomes and clinical responses in patients with severe pneumonia. Infection Chemotherapy. (2013) 45. doi: 10.3947/ic.2013.45.3.283 24396629 PMC3848515

[146] Alshehail BM Alwezzeh MJ Almalki HH Alnimr A Wali H Al Jamea Z . Recurrence, readmission, and key mortality predictors in patients with carbapenem-resistant Enterobacterales infections. Diagnostics. (2025) 15. doi: 10.3390/diagnostics15232957 41374338 PMC12691276

[147] Smith AMS Rockett RJ Oftadeh S Sintchenko V . Genomic features of Streptococcus pneumoniae associated with recurrent invasive pneumococcal disease. Pathology. (2026) 58:89–96. doi: 10.1016/j.pathol.2025.08.012 41314905

[148] Parsons JB Sidders AE Velez AZ Hanson BM Angeles-Solano M Ruffin F . In-patient evolution of a high-persister Escherichia coli strain with reduced *in vivo* antibiotic susceptibility. Proc Natl Acad Sci. (2024) 121:e2314514121. doi: 10.1073/pnas.2314514121 38190524 PMC10801923

[149] Peschel A Lin X-Q Liu Z-Z Zhou C-K Zhang L Gao Y . Trained immunity in recurrent Staphylococcus aureus infection promotes bacterial persistence. PloS Pathog. (2024) 20. doi: 10.1371/journal.ppat.1011918 PMC10798626 38241414

[150] Høiby N Moser C Ciofu O . Pseudomonas aeruginosa in the frontline of the greatest challenge of biofilm infection—its tolerance to antibiotics. Microorganisms. (2024) 12. doi: 10.3390/microorganisms12112115 PMC11596597 39597505

[151] Lodha R Puranik M Natchu U Kabra S . Recurrent pneumonia in children: clinical profile and underlying causes. Acta Paediatrica. (2002) 91:1170–3. doi: 10.1111/j.1651-2227.2002.tb00123.x 12463313

[152] Mei M Dai D Guo Z Zhang C Liu J Qi Y . Underlying causes and outcomes of recurrent pneumonia in hospitalized children. Pediatr Pulmonology. (2023) 58:1674–82. doi: 10.1002/ppul.26374 36919525

[153] Teramoto S Fukuchi Y Sasaki H Sato K Sekizawa K Matsuse T . High incidence of aspiration pneumonia in community- and hospital-acquired pneumonia in hospitalized patients: a multicenter, prospective study in Japan. J Am Geriatrics Soc. (2008) 56:577–9. doi: 10.1111/j.1532-5415.2008.01597.x 18315680

[154] Miyagami T Nishizaki Y Imada R Yamaguchi K Nojima M Kataoka K . Dental care to reduce aspiration pneumonia recurrence: a prospective cohort study. Int Dental J. (2024) 74:816–22. doi: 10.1016/j.identj.2023.11.010 38220512 PMC11287086

[155] Noguchi S Yatera K Kato T Chojin Y Fujino Y Akata K . Impact of the number of aspiration risk factors on mortality and recurrence in community-onset pneumonia. Clin Interventions Aging. (2017) 12:2087–94. doi: 10.2147/cia.s150499 29263657 PMC5724415

[156] Paris R . SARS-CoV-2 infection and response to COVID-19 vaccination in patients with primary immunodeficiencies. J Infect Dis. (2023) 228:S24–33. doi: 10.1093/infdis/jiad145 37539759 PMC10401615

[157] Aranda CS Gouveia‐Pereira MP da Silva CJM Rizzo MCFV Ishizuka E de Oliveira EB . Severe combined immunodeficiency diagnosis and genetic defects. Immunol Rev. (2024) 322:138–47. doi: 10.1111/imr.13310 38287514

[158] Grenier PA Brun AL Longchampt E Lipski M Mellot F Catherinot E . Primary immunodeficiency diseases of adults: a review of pulmonary complication imaging findings. Eur Radio. (2023) 34:4142–54. doi: 10.1007/s00330-023-10334-7 37935849 PMC11166740

[159] Schussler E Beasley MB Maglione PJ . Lung disease in primary antibody deficiencies. J Allergy Clin Immunology: In Pract. (2016) 4:1039–52. doi: 10.1016/j.jaip.2016.08.005 27836055 PMC5129846

[160] Chinnapaiyan S Dutta R Bala J Parira T Agudelo M Nair M . Cigarette smoke promotes HIV infection of primary bronchial epithelium and additively suppresses CFTR function. Sci Rep. (2018) 8:7984. doi: 10.1038/s41598-018-26095-z 29789655 PMC5964097

[161] Gavalda M Lorenzo A Vilchez H Gimenez S Calvo C Martin L . Skin lesions by Scedosporium apiospermum and Nocardia pulmonary infection in an oncologic patient: a case report. BMC Infect Dis. (2023) 23:523. doi: 10.1186/s12879-023-08484-6 37559001 PMC10413544

[162] Qi X Yang J Jin H Xiao Y Wang Y Zhang Y . Clinical characteristics and risk factors of connective tissue disease complicated with bronchiectasis and pulmonary infection. Apmis. (2024) 132:571–80. doi: 10.1111/apm.13424 38798084

[163] Gupta S Ferrada MA Hasni SA . Pulmonary manifestations of primary Sjögren's syndrome: underlying immunological mechanisms, clinical presentation, and management. Front Immunol. (2019) 10. doi: 10.3389/fimmu.2019.01327 31249572 PMC6583234

[164] Soto-Cardenas M-J Perez-De-Lis M Bove A Navarro C Brito-Zerón P Diaz-Lagares C . Bronchiectasis in primary Sjögren’s syndrome: prevalence and clinical significance. Clin Exp Rheumatol. (2010) 28:647–53. 20883638

[165] Løkke A Dongo LC Aksglæde KB Hilberg O . Asymptomatic dysphagia causing recurrent aspiration pneumonia. BMJ Case Rep. (2018) 2018. doi: 10.1136/bcr-2018-224370 PMC6109717 30131410

[166] Serel Arslan S Demir N Karaduman AA . Both pharyngeal and esophageal phases of swallowing are associated with recurrent pneumonia in pediatric patients. Clin Respir J. (2018) 12:767–71. doi: 10.1111/crj.12592 27925397

[167] Cao J Peng Z Lin H Huang Z Liu Z Pu Q . Prophylactic fiberoptic bronchoscopy after sleeve lobectomy can reduce the incidence of postoperative pneumonia: a propensity score matching study. J Thorac Dis. (2024) 16:8503–12. doi: 10.21037/jtd-24-1326 39831212 PMC11740041

[168] Zhao Y Zheng R Xiang W Ning D Li Z . Systematic review and meta-analysis on perioperative intervention to prevent postoperative atelectasis complications after thoracic surgery. Ann Palliative Med. (2021) 10:10726–34. doi: 10.21037/apm-21-2441 34763434

[169] Amour J Cholley B Ouattara A Longrois D Leprince P Fellahi J-L . The effect of local anesthetic continuous wound infusion for the prevention of postoperative pneumonia after on-pump cardiac surgery with sternotomy: the STERNOCAT randomized clinical trial. Intensive Care Med. (2019) 45:33–43. doi: 10.1007/s00134-018-5497-x 30617461

[170] Wiersinga WJ Rhodes A Cheng AC Peacock SJ Prescott HC . Pathophysiology, transmission, diagnosis, and treatment of coronavirus disease 2019 (COVID-19): a review. Jama. (2020) 324:782–93. doi: 10.1001/jama.2020.12839 32648899

[171] Deretic V Timmins GS . Enhancement of lung levels of antibiotics by ambroxol and bromhexine. Expert Opin Drug Metab Toxicol. (2019) 15:213–8. doi: 10.1080/17425255.2019.1578748 30721101 PMC6947664

[172] Tarique AA Tuladhar N Kelk D Begum N Lucas RM Luo L . Azithromycin augments bacterial uptake and anti-inflammatory macrophage polarization in cystic fibrosis. Cells. (2024) 13. doi: 10.3390/cells13020166 38247856 PMC10813867

[173] Bacharier LB Guilbert TW Mauger DT Boehmer S Beigelman A Fitzpatrick AM . Early administration of azithromycin and prevention of severe lower respiratory tract illnesses in preschool children with a history of such illnesses. Jama. (2015) 314. doi: 10.1001/jama.2015.13896 26575060 PMC4757487

[174] Haydar D Cory TJ Birket SE Murphy BS Pennypacker KR Sinai AP . Azithromycin polarizes macrophages to an M2 phenotype via inhibition of the STAT1 and NF-κB signaling pathways. J Immunol. (2019) 203:1021–30. doi: 10.4049/jimmunol.1801228 31263039 PMC6684391

[175] Shivaee A Kazemi F Navidifar T Golab N Parvizi E Mofid M . Global trends in macrolide and lincosamide resistance in Streptococcus species: a comprehensive systematic review and meta-analysis. BMC Infect Dis. (2025) 25. doi: 10.2139/ssrn.5393081 PMC12619386 41239271

[176] Iqbal F Derouen A Ren R Kaye AM Ahmadzadeh S Shekoohi S . Macrolide antibiotic mediated cardiac arrhythmias: emerging concepts and clinical implications. Biomedicines. (2025) 13. doi: 10.3390/biomedicines13061478 40564197 PMC12191034

[177] Wang Z Gan G Yao H . Gastrointestinal safety of oral erythromycin, clarithromycin, and azithromycin in pediatric patients: a FAERS pharmacovigilance study. Pediatr Res. (2025) 99:1051–9. doi: 10.1038/s41390-025-04312-6 40702206

[178] Comité de Neumonología . Bronchiectasis not related to cystic fibrosis in children: Guidelines for diagnosis, monitoring and treatment. Arch Argent Pediatr. (2020) 118:S164–82. doi: 10.5546/aap.2020.S164 33231063

[179] Zelaya H Villena J Lopez AG Alvarez S Agüero G . Modulation of the inflammation–coagulation interaction during pneumococcal pneumonia by immunobiotic Lactobacillus rhamnosus CRL1505: role of Toll‐like receptor 2. Microbiol Immunol. (2014) 58:416–26. doi: 10.1111/1348-0421.12163 24888715

[180] Mifsud EJ Kuba M Barr IG . Innate immune responses to influenza virus infections in the upper respiratory tract. Viruses. (2021) 13. doi: 10.3390/v13102090 34696520 PMC8541359

[181] Garcia-Castillo V Tomokiyo M Raya Tonetti F Islam MA Takahashi H Kitazawa H . Alveolar macrophages are key players in the modulation of the respiratory antiviral immunity induced by orally administered Lacticaseibacillus rhamnosus CRL1505. Front Immunol. (2020) 11. doi: 10.3389/fimmu.2020.568636 33133080 PMC7550464

[182] Albarracin L Dentice Maidana S Fukuyama K Elean M Argañaraz Aybar JN Suda Y . Orally administered lactobacilli strains modulate alveolar macrophages and improve protection against respiratory superinfection. Biomolecules. (2024) 14. doi: 10.3390/biom14121600 39766307 PMC11674826

[183] Kitazawa H Villena J . Modulation of respiratory TLR3-anti-viral response by probiotic microorganisms: lessons learned from Lactobacillus rhamnosus CRL1505. Front Immunol. (2014) 5. doi: 10.3389/fimmu.2014.00201 24860569 PMC4026741

[184] Wang Y Li X Ge T Xiao Y Liao Y Cui Y . Probiotics for prevention and treatment of respiratory tract infections in children. Medicine. (2016) 95. doi: 10.1097/md.0000000000004509 27495104 PMC4979858

[185] Torres A Cilloniz C Aldea M Mena G Miró JM Trilla A . Adult vaccinations against respiratory infections. Expert Rev Anti-infective Ther. (2025) 23:135–47. doi: 10.1080/14787210.2025.2457464 39849822

[186] Ale H Calderon JG Gruber J Taylor T Blouin WR Hernández-Trujillo VP . Pneumococcal serotype-specific antibodies in children with recurrent oto-sinopulmonary infections. Clin Exp Immunol. (2025) 219. doi: 10.1093/cei/uxae086 PMC11771189 39387142

[187] Hedlund J Ortqvist A Konradsen HB Kalin M . Recurrence of pneumonia in relation to the antibody response after pneumococcal vaccination in middle-aged and elderly adults. Scandinavian J Infect Dis. (2000) 32:281–6. doi: 10.1080/00365540050165929 10879599

[188] Loubet P Fourati S Launay O . RSV vaccination in immunocompromised adults. Int J Infect Dis. (2026) 169. doi: 10.1016/j.ijid.2026.108784 42119657

[189] Akikusa J Kemp A . Clinical correlates of response to pneumococcal immunization. J Paediatrics Child Health. (2001) 37:382–7. doi: 10.1046/j.1440-1754.2001.00687.x 11532059

[190] Chen J Lin W Yang C Lin W Cheng X He H . Immunogenicity, safety, and protective efficacy of mucosal vaccines against respiratory infectious diseases: a systematic review and meta-analysis. Vaccines. (2025) 13. doi: 10.3390/vaccines13080825 40872912 PMC12389829

[191] Li J Wu G Huang Z Hu J Tie X Wu H . Advances and prospects of respiratory mucosal vaccines: mechanisms, technologies, and clinical applications. NPJ Vaccines. (2025) 10. doi: 10.1038/s41541-025-01280-0 41224752 PMC12612081

[192] Vicente C Froes F Lopalco PL Heffernan C Nilforooshan R Martin F . From burden to benefit: overcoming barriers to immunization in adults, with a focus on respiratory syncytial virus. Expert Rev Vaccines. (2026) 25. doi: 10.1080/14760584.2026.2684962 42257651

[193] Scott J Abers MS Marwah HK McCann NC Meyerowitz EA Richterman A . Updated evidence for Covid-19, RSV, and influenza vaccines for 2025–2026. N Engl J Med. (2025) 393:2221–42. doi: 10.1056/nejmsa2514268 41160817

[194] McShane PJ . Investigation and management of bronchiectasis in nontuberculous mycobacterial pulmonary disease. Clin Chest Med. (2023) 44:731–42. doi: 10.1016/j.ccm.2023.07.005 37890912

[195] Košutova P Mikolka P . Aspiration syndromes and associated lung injury: incidence, pathophysiology and management. Physiol Res. (2021) 70:S567–83. doi: 10.33549/physiolres.934767 PMC9054185 35199544

[196] Bao L Zhang C Dong J Zhao L Li Y Sun J . Oral microbiome and SARS-CoV-2: beware of lung co-infection. Front Microbiol. (2020) 11:1840. doi: 10.3389/fmicb.2020.01840 32849438 PMC7411080

[197] Saint-Criq V Lugo-Villarino G Thomas M . Dysbiosis, malnutrition and enhanced gut-lung axis contribute to age-related respiratory diseases. Ageing Res Rev. (2021) 66:101235. doi: 10.1016/j.arr.2020.101235 33321253

[198] Putot A Garin N Rello J Prendki V . Comprehensive management of pneumonia in older patients. Eur J Intern Med. (2025) 135:14–24. doi: 10.1016/j.ejim.2025.02.025 40021428

[199] Åstrand A Kiddle SJ Siva Ganesh Mudedla R Porwal S Chafekar K Agrawal S . Effect of COVID-19 on bronchiectasis exacerbation rates: A retrospective U.S. insurance claims study. Ann Am Thorac Soc. (2024) 21:261–70. doi: 10.1513/AnnalsATS.202211-944OC PMC10848910 37962905

[200] Martínez-Vergara A Girón Moreno RM Olveira C Victoria Girón M Peláez A Ancochea J . Impact of the SARS-CoV-2 virus pandemic on patients with bronchiectasis: A multicenter study. Antibiotics (Basel). (2022) 11. doi: 10.3390/antibiotics11081096 PMC9405473 36009967

[201] Lyons PG Kollef MH . Prevention of hospital-acquired pneumonia. Curr Opin Crit Care. (2018) 24:370–8. doi: 10.1097/mcc.0000000000000523 30015635

[202] Bender RG Sirota SB Swetschinski LR Dominguez R-M Novotney A Wool EE . Global, regional, and national incidence and mortality burden of non-COVID-19 lower respiratory infections and aetiologies, 1990–2021: a systematic analysis from the Global Burden of Disease Study 2021. Lancet Infect Dis. (2024) 24:974–1002. doi: 10.1016/s1473-3099(24)00176-2 38636536 PMC11339187

[203] Vega V Rodríguez S Van der Stuyft P Seas C Otero L . Recurrent TB: a systematic review and meta-analysis of the incidence rates and the proportions of relapses and reinfections. Thorax. (2021) 76:494–502. doi: 10.1136/thoraxjnl-2020-215449 33547088 PMC8225554

[204] Iroh Tam P-Y Sadoh AE Obaro SK . A meta-analysis of antimicrobial susceptibility profiles for pneumococcal pneumonia in sub-Saharan Africa. Paediatrics Int Child Health. (2017) 38:7–15. doi: 10.1080/20469047.2017.1298700 28453405

[205] Zar HJ . Chronic lung disease in human immunodeficiency virus (HIV) infected children. Pediatr Pulmonology. (2007) 43:1–10. doi: 10.1002/ppul.20676 18041077

[206] Rodríguez L Cervantes E Ortiz R . Malnutrition and gastrointestinal and respiratory infections in children: A public health problem. Int J Environ Res Public Health. (2011) 8:1174–205. doi: 10.3390/ijerph8041174 PMC3118884 21695035

[207] Osei I Mendy E van Zandvoort K Sarwar G Nuredin IM Bruce J . Effect of the 13-valent pneumococcal conjugate vaccine on pneumococcal carriage in rural Gambia 10 years after its introduction: A population-based cross-sectional study. Vaccine. (2025) 56. doi: 10.2139/ssrn.5146327 40300436

[208] Ross I Bick S Ayieko P Dreibelbis R Wolf J Freeman MC . Effectiveness of handwashing with soap for preventing acute respiratory infections in low-income and middle-income countries: a systematic review and meta-analysis. Lancet. (2023) 401:1681–90. doi: 10.1016/s0140-6736(23)00021-1 37121242

[209] Bruce N . Indoor air pollution from unprocessed solid fuel use and pneumonia risk in children aged under five years: a systematic review and meta-analysis. Bull World Health Organ. (2008) 86:390–8. doi: 10.2471/blt.07.044529 18545742 PMC2647443

[210] Lai Y Chen B Chen S Shen Y . Experience of implementing metagenomic next-generation sequencing in patients with suspected pulmonary infection in clinical practice. Sci Rep. (2025) 15. doi: 10.1038/s41598-025-94840-2 40113957 PMC11926336

[211] Wu X Sun T He H Xing L Cheng Z Geng S . Effect of metagenomic next-generation sequencing on clinical outcomes of patients with severe community-acquired pneumonia in the ICU. Chest. (2025) 167:362–73. doi: 10.1016/j.chest.2024.07.144 39067508

[212] Quarton S Livesey A Jeff C Hatton C Scott A Parekh D . Metagenomics in the diagnosis of pneumonia: Protocol for a systematic review. JMIR Res Protoc. (2024) 13. doi: 10.2196/57334 39293053 PMC11447427

[213] Chen K Ma W Zhong J Yang P He N Li X . Probiotic supplementation reduces RRTIs and enhances gut microbial and immunity in children: A randomized controlled trial. J Microbiol Biotechnol. (2026) 36:e2511038. doi: 10.4014/jmb.2511.11038 41635177 PMC12883317

[214] Koenen MH de Steenhuijsen Piters WAA Bogaert D Verhagen LM . The microbiota in respiratory tract infections: From association to intervention. Curr Opin Infect Dis. (2022) 35:215–22. doi: 10.1097/qco.0000000000000826 35665715

[215] Nakahira A Iwaoka H Sato K Ishizuka S Shiroma R Miyasaka T . NKT cell–dependent PspA-specific IgA production caused by mucosal administration of a novel nanoparticle pneumococcal vaccine. Vaccine. (2025) 68. doi: 10.1016/j.vaccine.2025.127911 41175650

[216] Acosta D Rubel Hoq M Takeda K Costley CD Golding H Zaitseva M . Development of *in vitro* airway epithelial model to assess immune response and safety of mucosal adjuvants. NPJ Vaccines. (2026) 11. doi: 10.1038/s41541-026-01459-z 42014708 PMC13287757

[217] Morens DM Taubenberger JK Fauci AS . Rethinking next-generation vaccines for coronaviruses, influenzaviruses, and other respiratory viruses. Cell Host Microbe. (2023) 31:146–57. doi: 10.1016/j.chom.2022.11.016 36634620 PMC9832587

